# Application of asymmetric Sharpless aminohydroxylation in total synthesis of natural products and some synthetic complex bio-active molecules

**DOI:** 10.1039/c7ra12625e

**Published:** 2018-02-09

**Authors:** Majid M. Heravi, Tahmineh Baie Lashaki, Bahareh Fattahi, Vahideh Zadsirjan

**Affiliations:** Department of Chemistry, School of Science, Alzahra University Vanak Tehran Iran mmh1331@yahoo.com mmheravi@alzahra.ac.ir z_zadsirjan@yahoo.com

## Abstract

This report illustrates the applications of Asymmetric Sharpless Aminohydroxylation (ASAH) in the stereoselective synthesis of vicinal amino alcohols as important intermediates in the total synthesis of complex molecules and natural products with significant biological activities. The ASHA allows the regio- *syn*-selective synthesis of 1,2-amino alcohols *via* reaction of alkenes with salts of *N*-halosulfonamides, -amides and -carbamates employing osmium tetroxide (OsO_4_) as an efficient catalyst. In this reaction, chirality is induced *via* the addition of dihydroquinine- and dihydroquinidine as derived chiral ligands.

## Abstract 2

This work shows applications of Asymmetric Sharpless Aminohydroxylation (ASAH) in the stereoselective synthesis of vicinal amino alcohols as important intermediates in the total synthesis of complex molecules and natural products with significant biological activities.

## Graphical abstract

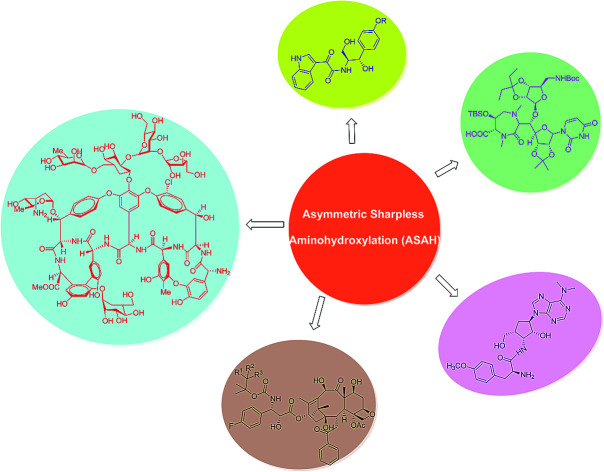

## 1. Introduction

Amino alcohols contain both an amine and an alcohol group. Enantiomerically pure amino alcohols play a progressively significant role in pharmaceutical therapy.^1^ β-Amino alcohols can be used as chiral auxiliaries in asymmetric synthesis.^2^ Amino alcohol derivatives are currently being studied for their antimicrobial and antifungal activities, and in the modulation of the physiochemical properties of drug molecules.^3^ The commercial availability or easy accessibility of amino alcohols makes them an appealing class of versatile promoters to exploit in modern organic synthesis.^4,5^ Significantly, they are frequently used as pharmacophores in drug discovery, thus their asymmetric synthesis has always been in much demand.

The *vic*-amino alcohol moiety can be provided by coupling of two units, one containing the oxygen functionality and the other one containing the nitrogen functionality, with a simultaneous formation of a new carbon–carbon bond involving vicinal chiral centers that requires both enantio- and diastereo control. Generally, this approach is limited to certain types of substrates. This approach can be achieved *via* Mannich-type reactions,^6^ that one sophisticated example on a highly stereoselective Mannich-type reaction is based on a nucleophilic additions of α-alkoxy enolates to imines affording amino alcohols with high to excellent yields.^7^

The *vic*-amino alcohols could also be synthesized employing Lewis acid-catalyzed aldol reactions. Zirconium/BINOL-catalyzed reactions of glycine derived silyl ketene acetals to aldehydes furnishes *anti*-β-hydroxy-α-amino acids in excellent yields and enantioselectivities.^8^

Another approach is to utilize the stereo directing influence of a preexisting chiral center. This can be achieved by nucleophilic additions to α-amino aldehydes, which often proceed with good diastereoselectivity. An example, is a divergent strategy for substrate-controlled diastereoselective synthesis of aminodiols based on nucleophilic Mukaiyama aldol additions to α-amino-β-silyloxy aldehydes.^9^ The most direct approach toward enantioselective synthesis of *vic*-amino alcohols is the asymmetric Sharpless aminohydroxylation (ASAH) of alkenes. The chiral catalyst utilized in this reaction is the same as in the asymmetric Sharpless dihydroxylation reaction (ASDH). α,β-Unsaturated esters and phosphonates have proven to be the most appropriate substrates for this reaction. Although this transformation is an attractive approach to the direct enantioselective synthesis of amino alcohols, the yields are frequently moderate perhaps due to regioselectivity complications.^10^

ASAH features the *syn*-selective synthesis of 1,2-amino alcohols through treatments of alkenes with salts of *N*-halosulfonamides, -amides and -carbamates in the presence of osmium tetroxide as a catalyst. Moreover, chirality is induced *via* the addition of chiral ligands such as dihydroquinine- and dihydroquinidine.^11^ ASAH reaction offers practically direct access to the collection of amino alcohols, which is present in several biologically active complex molecules and naturally occurring compound.^12^

Consequently, the ASAH reaction speedily obtained the eminence of its other K. B. Sharpless forerunners such as, the asymmetric Sharpless epoxidation (ASE)^13^ and asymmetric Sharpless dihydroxylation (ASD)^14^ strategies, thus belongs to the important other pieces of work already presented by Sharpless and co-workers for which in 2001, he was introduced as a Nobel Prize Laureate in Chemistry.

**Scheme 1:**
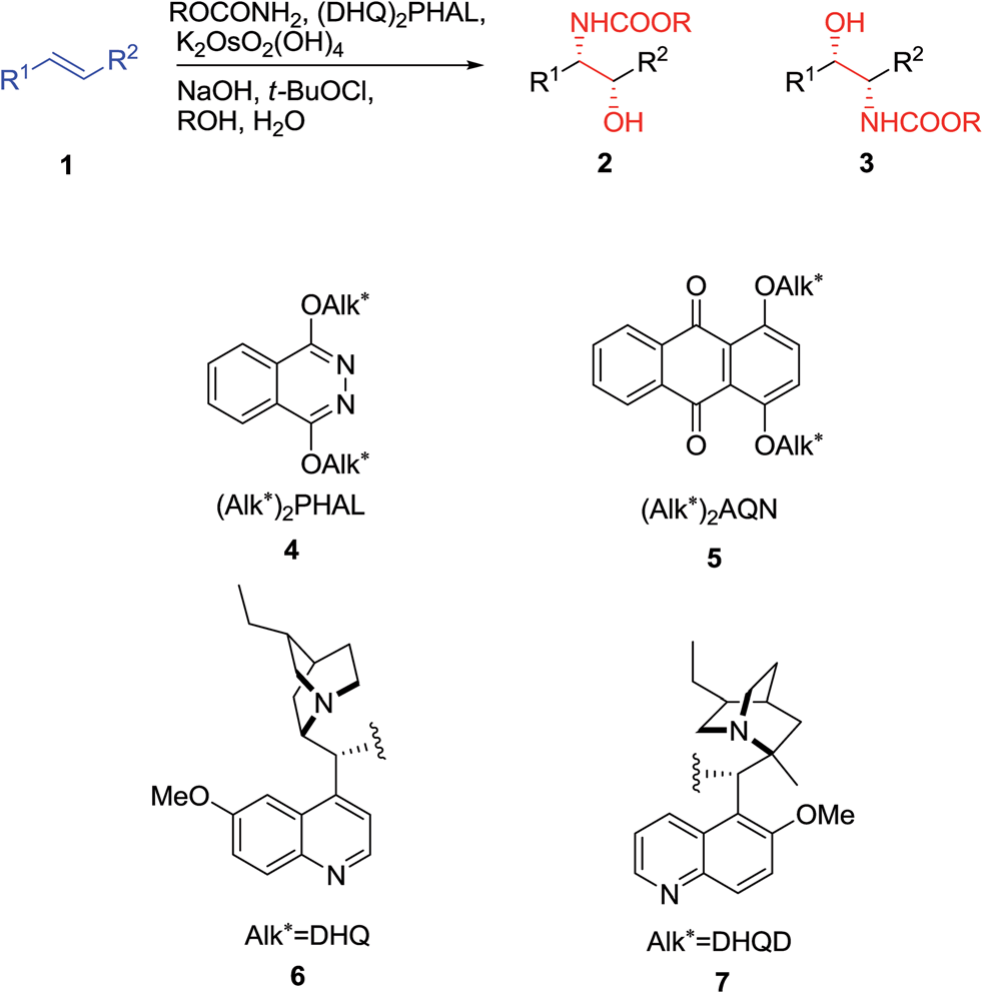


The reaction, symbolized by the transformation, exhibited in Scheme 1. In this reaction, a complex involving Cinchona alkaloid derived ligands with an osmium species as an oxidant in amalgamation with a stoichiometric nitrogen source is used. A broad range of nitrogen sources are accessible that only differ in the *N*-substituent with each other, thus providing inversely protected amino alcohols. The protecting groups used are usually *t*-butoxycarbonyl (Boc), benzyloxycarbonyl (Cbz), and 2-(trimethylsilyl)ethoxycarbonyl (Teoc).^15^

The chiral ligands can induce chirality, leading to enantioselectivity in the products that happens by preference addition to one enantiotopic face of the pro-chiral of an alkene as substrate. As an example, the 1,4-bis(9-*O*-dihydroquininyl)-phthalazine [(DHQ)_2_PHAL] as ligand directs addition to the α-face of an alkene 1 to give amino alcohol as products 2 or 3 (Scheme 1) whereas, the 1,4-bis(9-*O*-dihydroquinidinyl)-phthalazine [(DHQD)_2_PHAL] ligand guides addition to the β-face of 1. Significantly, the sense of enantioselective induction meticulously matches that detected in the ASDH reaction, proposing that the similar parameters overriding the ee values both in ASD and ASAH process.^15^ Noticeably, an additional complication relative to ASDH, which can be arisen, is that in the ASAH reaction the regioselectivity is also an issue. For example, the oxidation of unsymmetrical alkenes 1 (R^1^ ≠ R^2^), basically can lead to the formation in two regioisomeric amino alcohol, products 2 and 3. Frequently, the aromatic linker of the chiral ligand or in the reaction conditions of for instance when phthalazine (PHAL) or anthraquinone (AQN) are used can strongly affect the regioselectivity of the reaction (Scheme 1).^16^

It should be seriously noticed that the control of regioselectivity is habitually the highest challenge in the application of the ASAH reaction in organic and especially in the total synthesis of natural products. Noticeably, in spite of the vast potential of this reaction, in the early years of introduction of ASAH reaction only relatively few researchers showed interest to develop such an important synthetic approach. Also, it was largely overlooked to be used as a key step in the total synthesis of natural products. Probably, this lack of interest was due to the challenging the problem of controlling of both the regio- and enantioselectivity of the ASAH reaction when the complex molecules are being used as substrates. However in recent years, several efficient approaches for circumventing such problems have been attempted and opened new gate away for the effective and facile synthesis of amino alcohols being used as precursors in a crucial step of the complex molecules and natural products comprising these important motifs.^15^

We are interested in asymmetric synthesis^17–25^ especially those applied to total synthesis of natural products.^18,26–34^ We recently published on the applications of Sharpless asymmetric epoxidation (SAE)^24^ and Sharpless asymmetric dihydroxylation (SAD)^20^ in the total synthesis of natural products. As a supplementary, in this report we try to underline the applications of asymmetric Sharpless aminohydroxylation (ASAH) in the synthesis of natural products showing biological properties. It should be mentioned that ASAH reaction has been reviewed previously, comprising the application of this reaction.^11,12,35–37^ It is worthy to mention that in 2002, the mechanism as well as applicability of ASAH in common synthetic tasks along with its scope and limitation were fully discussed.^38^ In this review, we only focus on ASHA reaction in the total synthesis of natural products as a supplementary to our previous reports.^20,24^ We have covered the applications ASAH reaction in the total synthesis of natural products and some complex molecules with significant biological activities, which have not been necessarily isolated from natural sources.

## 2. Mechanism

**Scheme 2:**
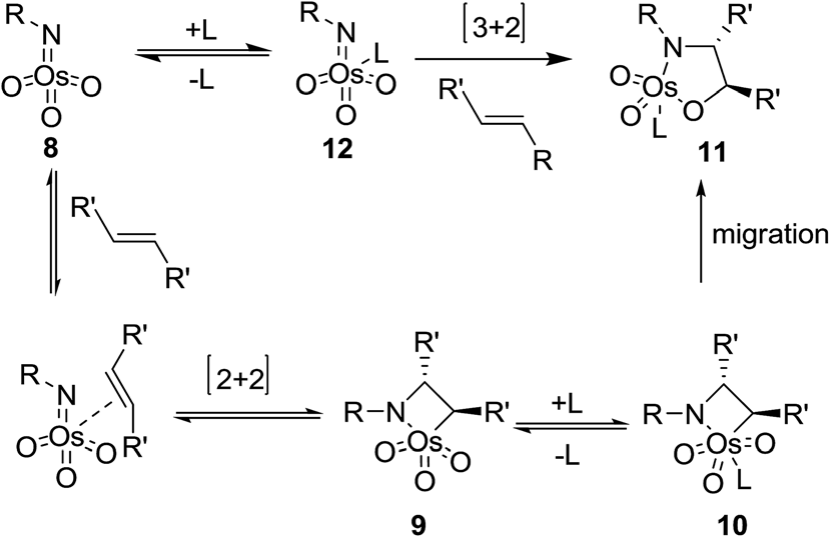


Two different mechanisms have been proposed for ASAH reaction, that both proposals are closely based on mechanistic investigations of its forerunner, the ASDH reaction. The first mechanism was proposed by Sharpless.^39^ It actually counterparts the mechanism suggested for ASDH reaction.^40^ It involves a classical [2 + 2] cycloaddition of the alkene to the imido trioxoosmium species 8 generates the osmaazetidine 9, which subsequently coordinated to ligand to provide intermediate 10. The latter was subjected into 1,2-migration of the carbon–osmium bond to create the osmium azaglycolate 11 as an addition product. However, this mechanism was argued from electronic point of view. It has been found that nitrogen is often added to the β-carbon of alkenes attached to an electron withdrawing group, regioselectively.^39^ The advantage properties of the ligand on the enantio- and regioselectivity of ASAH reaction take place by inducing the position of the equilibrium thus, preferring one of the diastereomeric complexes denoted by 10 or by controlling the relative rate of ultimate bond migration to form 11 (Scheme 2).^41^

**Scheme 3:**
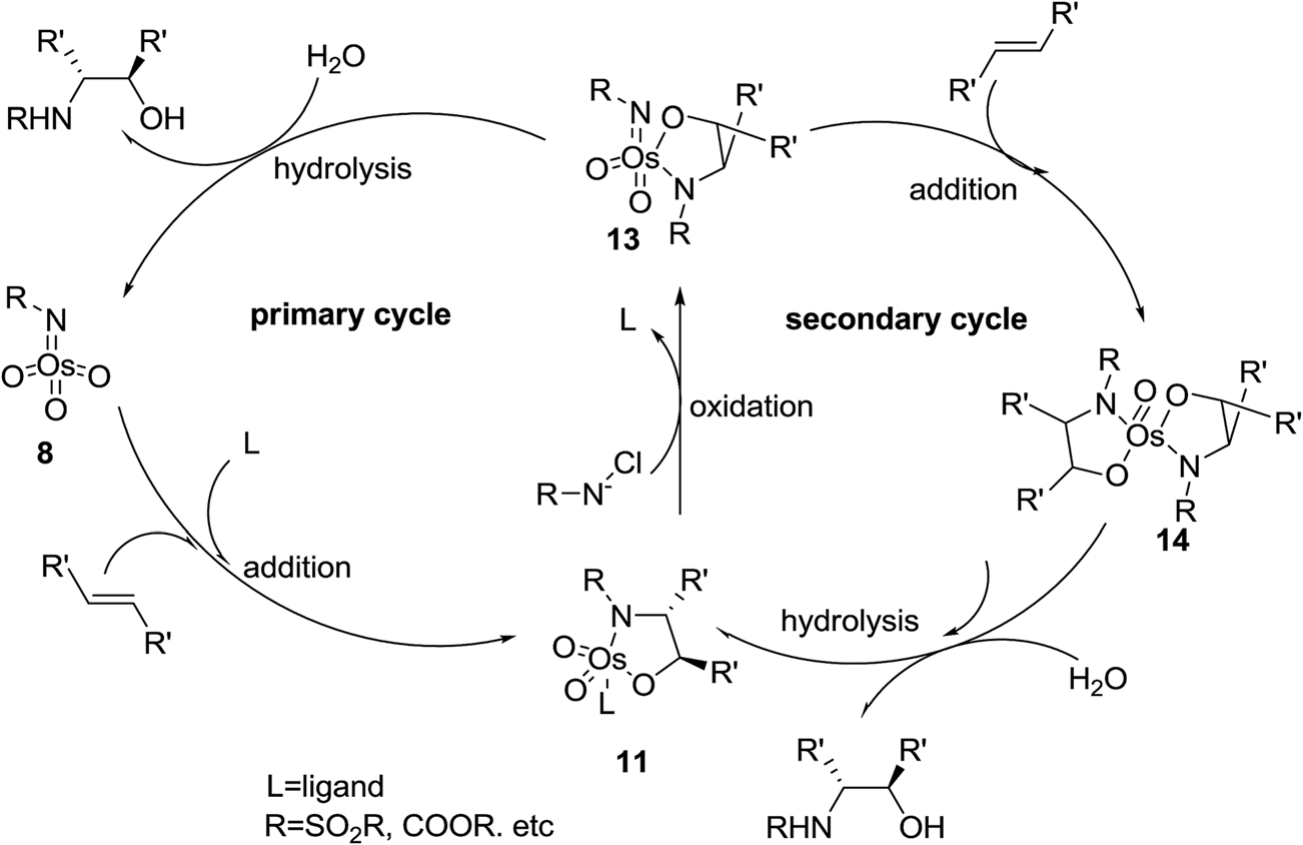


The second suggested mechanism involves^42^ the formal [3 + 2] cycloaddition of ligand-bound complex 12 to the alkene, similar to the Criegee mechanism for osmium-catalyzed dihydroxylation.^43^ In this occasion, it is assumed that the selectivity of the addition is controlled by catalyst–substrate interactions. However, serious dispute^44^ is enclosed for the related mechanism to the osmium-mediated ASDH reaction. Nevertheless, several recent conclusions, comprising kinetic isotope effects^45^ and computational studies,^45,46^ favors to the mechanism involving [3 + 2] cycloaddition reaction. This instance aside, the mechanistic route for the vital bond-formation step related to with ASAH reaction, which demands further study. Regardless of the precise mode of addition, it is observed that the ligand enhances the rate, affects regioselectivity and induces excellent enantioselectivity in the ASAH reaction. These observations led to the suggested mechanism illustrated in Scheme 3 it involves two catalytic cycles, each affording different incomes for selectivity and competing to afford different products.^39,47^ The first cycle is promoted by the alkaloid derived ligand and in all ASAH reaction reported till date except one.^48^ It is observed that ligand improves the catalytic transformation relative to the non-ligand-promoted reaction. Ligand-promoted addition of imidotrioxoosmium(viii) species 8 to the alkene generates azaglycolate species 11 (Scheme 3). The species 11 can be reoxidized by the nitrogen source to make 12, which upon hydrolysis is regenerating, 8 and release the product. Worthy to mention that alternatively, 11 can be hydrolyzed with subsequent oxidation to 8. The oxidised azaglycolate species 11 can also go in the secondary cycle, added to a second alkene affording bis(azaglycolate)osmium species 14. The addition step of this cycle is nothing to do with the Cinchona alkaloid derived ligand thus, expectedly affording addition products with low enantio- and regioselectivity. Hydrolysis of 14 results in the generation of 11 back which, re-entering to either the primary or the secondary cycle. The hydrolysis of azaglycolate complexes 13 or 14 (ref. 49) is conversion-determining step in both catalytic cycles. Control of the oxidation by performing the reaction in aqueous solvent mixtures is accomplished. Thus, hydrolysis of 13 is preferred leading to preference of the primary cycle.^39,47^ In this case, elimination of the secondary cycle was most efficiently achieved by performing the reaction in which the media involves biphasic aqueous-organic solution.^14^ The majority of ASAH reported so far have been performed under homogeneous conditions thus dominance of the secondary cycle bases on efficient hydrolysis of 13 (Scheme 3).

## 3. Application of asymmetric Sharpless aminohydroxylation (ASAH) in total synthesis of

### 3.1. Alkaloids

**Scheme 4:**
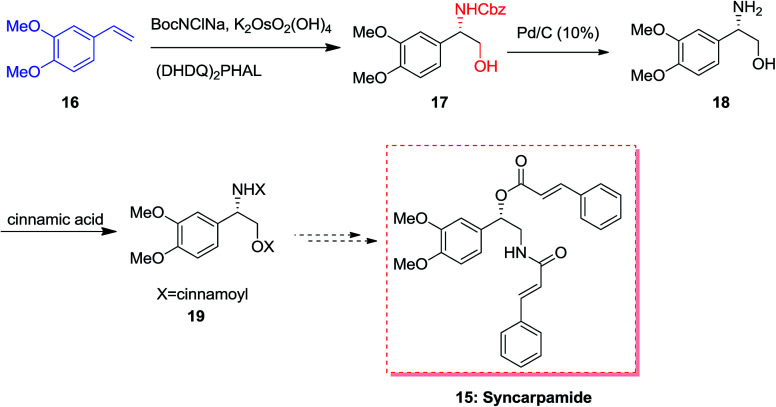


**Scheme 5:**
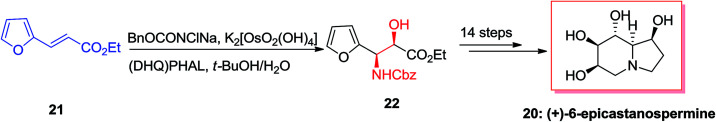


**Scheme 6:**
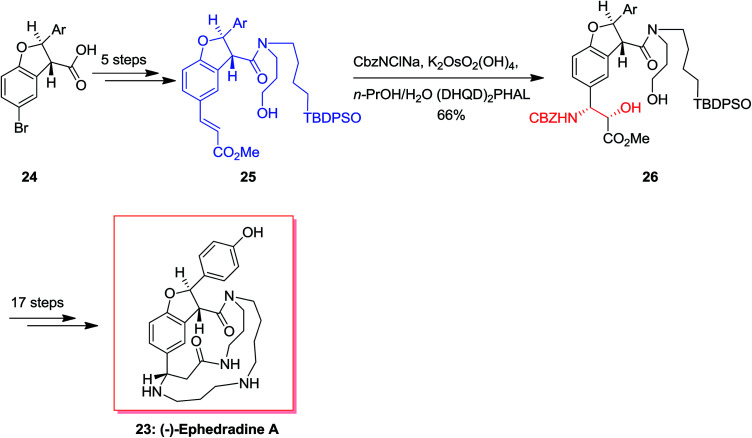


In 2004, Ross and co-workers^50,51^ demonstrated the isolation and antiplasmodial activity of norepinephrine alkaloid, syncarpamide 15 from the extract leaves of *Zanthoxylum syncarpum* (Rutaceae). Syncarpamide 15. This natural product showed antiplasmodial properties toward *P. falciparum*. In 2017, Bhattacharya and co-workers achieved and reported the total synthesis of analogues of syncarpamide 15. Notably, by considering the structure–activity relationship point of view, the total synthesis of a molecule bearing functional groups interchanged with reference to the parent molecule *i.e.* regioisomer 19 showing *vis-à-vis* syncarpamide 15 was contemplated. In this line, dimethoxy styrene 16 underwent ASAH^52^ providing compound 17 which upon Cbz deprotection using Pd/C (10%) completed the total synthesis of desired target amino alcohol 18 that was coupled with cinnamic acid to supply the desired regioisomer 19 of syncarpamide 15 (Scheme 4).^53^

(+)-6-Epicastanospermine 20, exist along with castanospermine was initially found in Australian legume is a potent inhibitor of amyloglucosidase (an *exo*-1,4-α-glucosidase).^54^ A highly effective stereoselective total synthesis of (+)-6-epicastanospermine 20 was established using the ASAH reaction of furyl acrylate 21 as an essential step. Remarkably, one of the striking aspects of this method based on its intrinsic flexibility is the stereoselectivity of ASAH reaction of furyl acrylate, which could be achieved by using various ligands. ASAH reaction of furyl acrylate 21 by employing (DHQ)_2_PHAL as the chiral ligand produced β-hydroxy-α-furfurylamine 22 in 87% ee and 62% yields.^55^ Finally, compound 22 was subjected to different reactions in 14 steps provided the desired natural product (+)-6-epicastanospermine 20 (Scheme 5).^55^

(−)-Ephedradine A (orantine, 23), a complex macrocyclic spermine alkaloid, was extracted by Hikino and co-workers in 1979 that found as one of the hypotensive components of the Chinese traditional drug “mao-kon.”^56–58^ For the total synthesis of (−)-ephedradine A 23, carboxylic acid 24 has been applied as starting compound that upon 11 steps gave the cinnamate 25. Significantly, simple and diastereoselective incorporation of the nitrogen atom in cinnamate 25 has been accomplished through the ASAH reaction to give 26 as the main product (12 : 1). Upon several steps, the desired natural product 23 was provided (Scheme 6).^59,60^

### 3.2. Amino alcohols

**Scheme 7:**
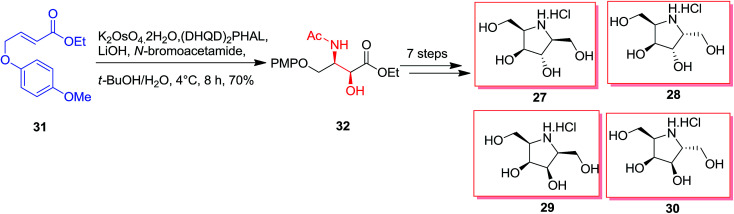


The essential vicinal amino alcohol group was provided regio- and enantioselectively using Os-mediated ASAH reaction of the planned achiral olefin.^61^ Total synthesis of polyhydroxylated pyrrolidine 27–30 was started from the olefin 31. The ASAH reaction of the olefin 31 by using (DHQD)_2_PHAL and *N*-bromoacetamide gave the *syn*-amino alcohol 32 with a high regioselectivity (>20 : 1) and ee (>99%), that after seven steps afforded the desired polyhydroxylated pyrrolidines 27–30 (Scheme 7).^62^

**Scheme 8:**
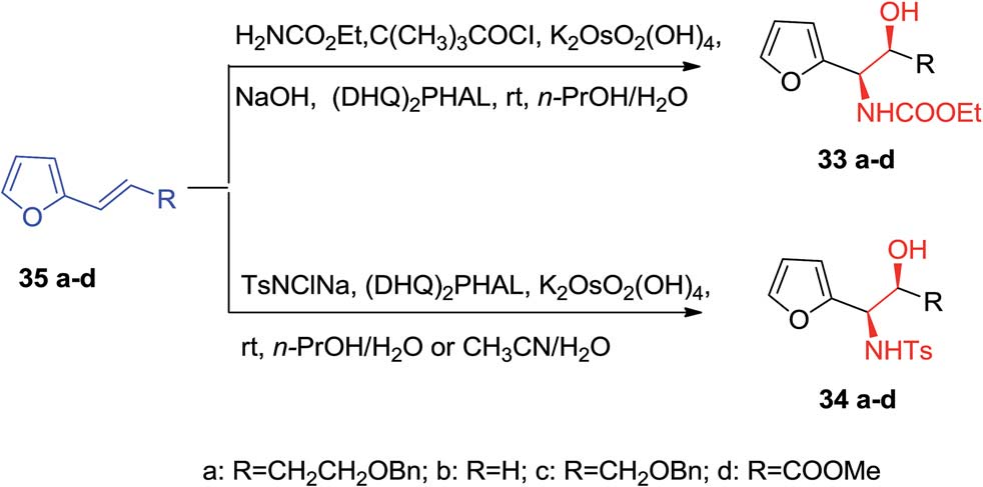


Optically active α-furfuryl amines have stimulated the attention of organic chemists in recent years, since they are extremely valuable building scaffolds for the formation of a significant range of nitrogen comprising naturally occurring compounds, including α-amino acids, indolizilines, β-lactams, piperidine and quinolizidines alkaloids.^63^ Significantly, ASAH reaction is a very valuable approach for providing both amino and hydroxy substituents directly to the olefins with excellent ee. ASAH reaction of α-furyl ethylenes 35a–d yielded the α-furfuryl amines 33a–d or 34a–d, that might be the valuable chiral building scaffolds for the construction of polyhydroxylated indolizidine alkaloids, for example castanospermine, that is a powerful inhibitor of glycosidase and glycoprotein processing (Scheme 8).^63,64^

**Scheme 9:**
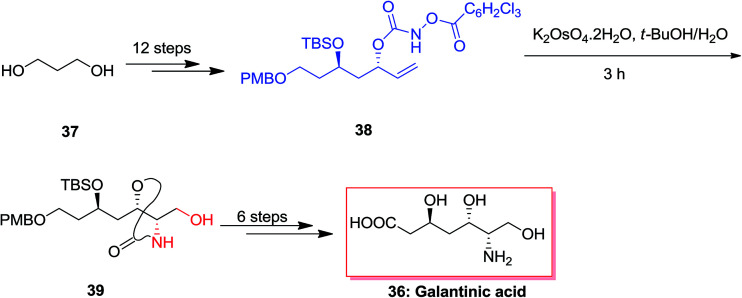


An efficient synthetic method was demonstrated for the synthesis of (−)-galantinic acid 36 by using iterative hydrolytic kinetic resolution and tethered ASAH reaction as the main stages. The synthesis of (−)-galantinic acid 36 was initiated from market purchasable 1,3-propanediol 37, that upon 12 steps yielded compound 38. Next, compound 38 was exposed to tethered ASAH reaction based on improved and normalized reaction conditions. Significantly, slow addition of K_2_OsO_4_·2H_2_O to the solution of 38 in *t*-BuOH/H_2_O provided the masked amino alcohol 39 in 75% yields. Finally, after six steps, (−)-galantinic acid 36 was synthesized in 1.52% overall yields (Scheme 9).^65^

**Scheme 10:**
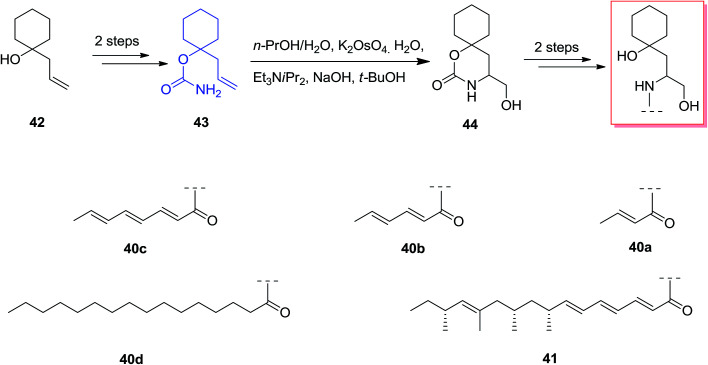


Scyphostatin was extracted from a mycelial extract of *Dasyscyphus mollissimus* SANK-13892 in 1997 by Ogita and co-workers and its gross structure identified by widespread spectroscopic and derivatisation studies.^66^ Kenworthy and co-workers described using the first tethered aminohydroxylation reaction using a tertiary alcohol as part of a route to synthesize analogues of the naturally occurring sphingomyelinase inhibitor, scyphostatin. The tethered aminohydroxylation of 1-allylcyclohexanol provides the β-amino alcohol product, in masked form, with the requisite regiochemistry. Total synthesis of β-amino alcohols was started from the known homo-allylic alcohol 42, which was provided in quantitative yields from cyclohexanone, was reacted with trichloroacetyl isocyanate. Next, the carbamate 43 was produced through methanolysis of the intermediate trichloroacetylcarbamate in high yield. In the following, reaction of 43 based on the usual TA conditions^67^ resulted in construction of cyclic carbamate 44 in 40% yield, with recovery of 45% of the starting compounds. Two more reactions has been used to provide the analogues 40a–d and 41 in satisfactory yield, with no protection required during the method (Scheme 10).^68^

**Scheme 11:**
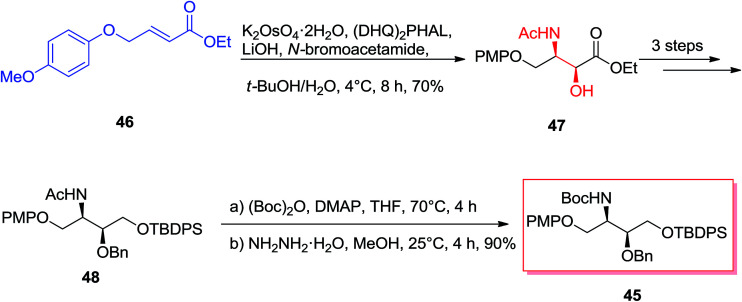


Enantiomerically vicinal amino alcohol derivatives make a significant group of natural and synthetic organic compounds.^69^ Among these, of particular attention are the orthogonally masked 2-amino-1,3,4-butanetriols (ABTs), meanwhile they can give useful four carbon chiral building blocks for the enantioselective synthesis of an extensive range of biologically significant organic compounds including anticancer agents, antiviral agents, and antibiotics.^70^ Theoretically, the most effective approach for the providing of the vicinal amino alcohol functionality and stereochemistry of ABTs would be through the ASAH reaction of suitable four carbon olefins. Singh and co-workers demonstrated a tremendously concise and stereoselective synthesis of the orthogonally masked ABTs.^71^ Enantioselective synthesis of the orthogonally masked *anti* and *syn*-ABTs was established that used the regioselective ASAH reaction of oxazoline and olefins chemistry. Moreover, since the enantiomers of 45 can be prepared by using (DHQ)_2_PHAL ligand in place of (DHQD)_2_PHAL for the regioselective ASAH reaction of 46, this method demonstrates a common solution for the complete enantioselective synthesis of ABTs from the easily accessible achiral olefin 46.^71^Scheme 11 illustrates synthesis of the orthogonally masked *syn*-ABTs initiating from the achiral α,β-unsaturated ester 46, that the regioselective ASAH reaction of 46 gave the *syn*-amino alcohol 47 with a high ee (>99%) and regioselectivity (>20 : 1). Remarkably, the 4-(*p*-methoxy)phenoxy group of in 46 shows a dual role in synthesis: its aryl–aryl stacking interaction with the aryl groups of the ASAH catalyst can improve enantio- and regioselectivity of the ASAH reaction of 46, and also it can serve as a suitable alcohol masking group (role as a protection group). Next, *syn*-amino alcohol 47 after several steps produced the *N*-acetyl group of 48, that was converted into the more readily removable and manipulable *N-t*-butyloxycarbonyl (Boc) group *via* reacting Boc anhydride and hydrazine hydrate in MeOH to yield 45. Significantly, the short enantioselective synthesis of the orthogonally masked *syn*-(2*R*,3*S*)-2-amino-1,3,4-butanetriol 48 has been performed in an overall 51.9% yields.^71^

**Scheme 12:**
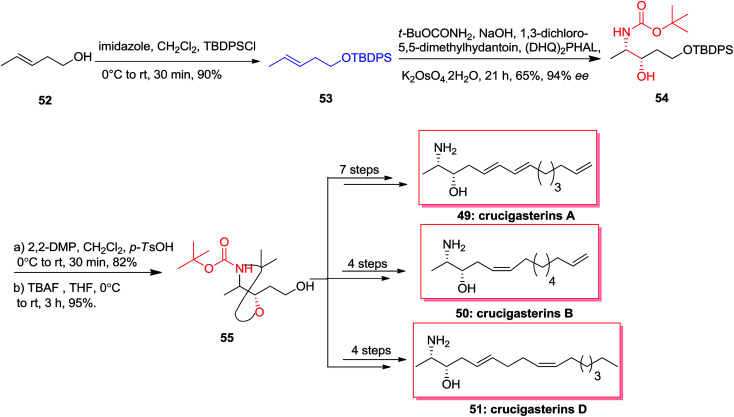


In 2013, the first stereoselective total synthesis of the marine-obtained antimicrobial amino-alcohol derivatives, crucigasterins A 49, B 50 and D 51 were accomplished initiating from pent-3-en-1-ol 52. This approach includes the ASAH reaction and Wittig olefinations as the essential stages. Total synthesis of crucigasterins A 49, B 50 and D 51 were started from the alcohol 52 that was transformed into its TBDPS ether 53 through reaction with imidazole and TBDPSCl. In the following, the double bond of compound 53 was exposed to ASAH reaction by (DHQ)_2_PHAL, *t*-BuOCONH_2_ and K_2_OsO_4_·2H_2_O to give amino alcohol 54. Next, aminoalcohol 54 was protected by 2,2-DMP and then the ether group was cut by TBAF to produce the intermediate 55. Finally and upon several steps, the natural products crucigasterins A 49, B 50 and D 51 were synthesized *via* different routes (Scheme 12).^72^

**Scheme 13:**
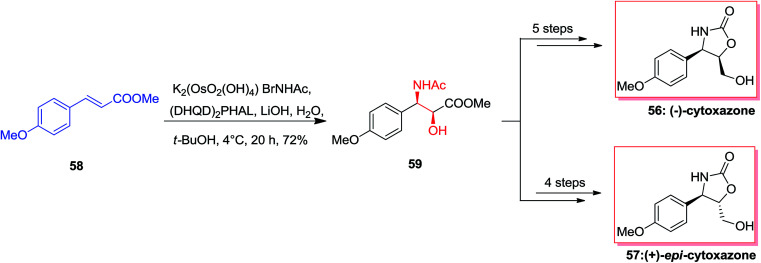


Cytoxazone 56, a natural occurring compound, was extracted in 1998 from a fermentation broth of *Streptomyces* sp.^73^ Total synthesis of optically pure (−)-cytoxazone and (+)-*epi*-cytoxazone 57 have been reported by Milicevic and co-workers initiating from easily accessible methyl *p*-methoxycinnamate 58. The desired *anti*-amino alcohol 59 configuration was developed by mixing ASAH reaction and the configurational inversion of the intermediate amido alcohol through an oxazoline. For the synthesis of (−)-cytoxazone 56, ASAH reaction of 58 with (DHQD)_2_PHAL afforded the corresponding amido alcohol 59 in 72% yield. Remarkably, optically pure (−)-cytoxazone 56 was prepared in six steps and in 31% overall yield. Furthermore, in a similar way, (+)-*epi*-cytoxazone 57 was synthesized from 58, in five steps and 36% overall yields (Scheme 13).^74^

**Scheme 14:**
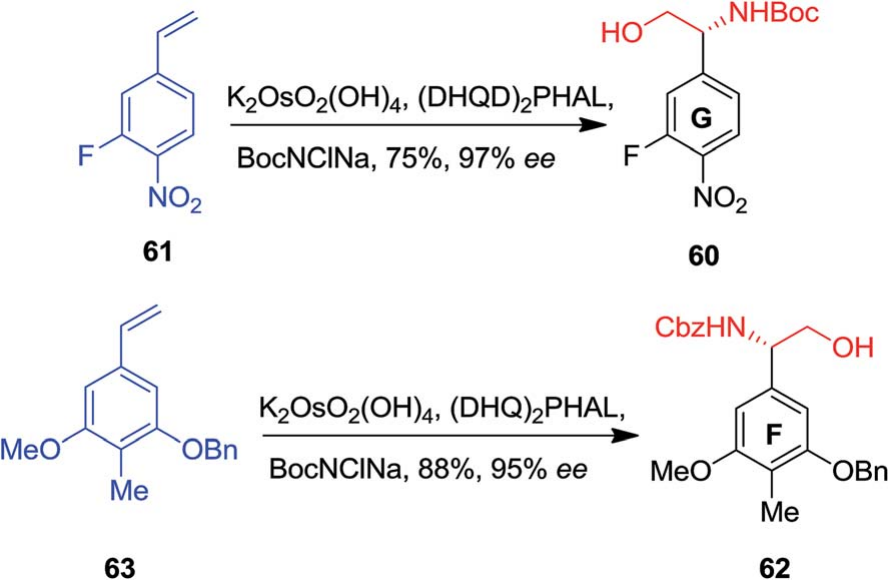


In 2004, Boger and co-workers reported the first total synthesis of the ristocetin aglycon using a modular and extremely convergent method.^52^ In this approach, for the synthesis of the F and G ring phenylglycine precursors, ASAH reaction were used as the key step. The G ring precursor 60, an (*R*)-phenylglycinol, has been provided from vinylbenzene 61 that after ASAH reaction yielded (*R*)-phenylglycinol 60 in 75% yields and 97% enantioselectivity. Remarkably, the F ring (*S*)-phenylglycinol 62, has been produced from nitro vinylbenzene 63 and ASAH reaction in the presence of CbzNClNa resulted in (*S*)-phenylglycinol 62 in 88% yields and 95% enantioselectivity (Scheme 14).^52^

**Scheme 15:**
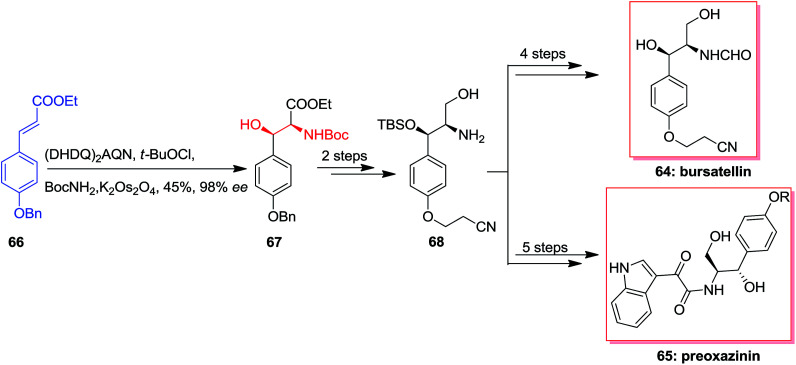


In 2006 Cimminello and co-workers reported isolation of oxazinin-4 from toxic mussels. Several members of this group was extracted and some of the members such as oxazinin-1 has shown biological properties.^75^ Due to lack of toxicological studies of oxazinines and restriction of isolation, several attempts has been done to form different members of this group.^76,77^ In this route, Dethe and co-workers in 2013 reported total synthesis of preoxazinin 65 and bursatellin 64. Total synthesis of them was initiated from phenyl acrylate 66, which using ASAH afforded directly Boc protected amino alcohol 67 in 45% yields and 98% enantioselectivity. Next, masked amino alcohol 67, upon some steps, resulted in Boc-deprotected amine 68 in high yields. In the following, amine 68 through different pathways generated bursatellin 64 in 80% yield and also, the natural product preoxazinin 65 (Scheme 15).^78^

### 3.3. Amino acids and peptids

**Scheme 16:**
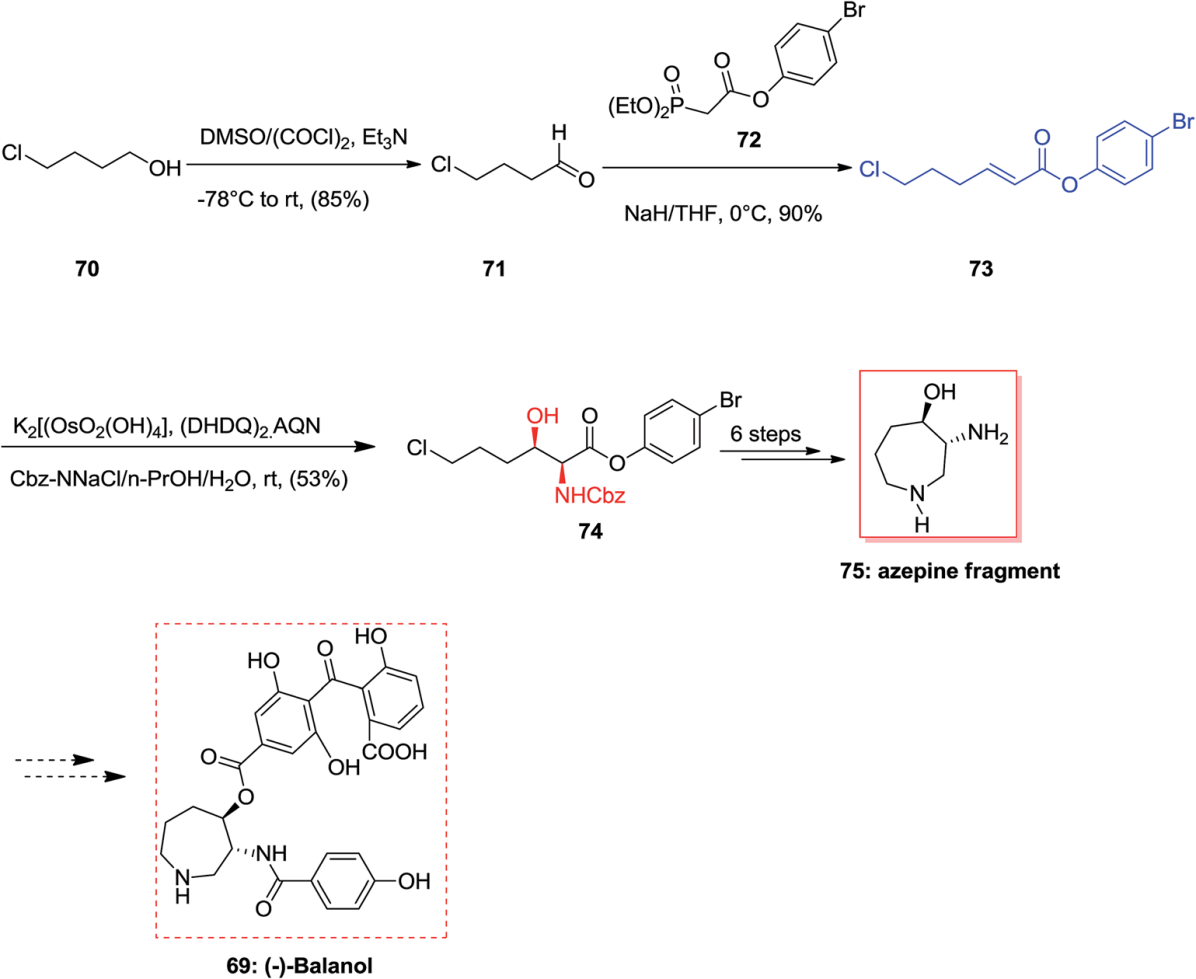


(−)-Balanol 69, an uncommon metabolite, was initially extracted from the fungus Verticillium balanoides^79^ in 1993. However, then again in 1994 compound 69 was extracted and isolated from different fungus, *Fusarium merismoides*.^80^ Compound 69 showed being a strong inhibitor of human protein kinase C (PKC). Latter, this enzyme group showed an essential role in signal transduction routes which result in a range of cellular responses involving gene expression and cellular proliferation.^81^ Therefore, this class of enzymes were considered as significant and a vital and potent biologically active target for the designing of anticancer drugs and therapeutic agents to control inflammation, as well as central nervous system disarrays, cardiovascular disorders, and even to heal HIV infection. An outstanding strategy towards the concise formal total synthesis of the active protein kinase C inhibitor (−)-balanol 69 was achieved and reported by Panek and co-workers in 2000. This protocol relies on a modified ASAH of the α,β-unsaturated aryl ester. The aryl ester functionality and the dihydroquinyl alkaloid ligand system (DHQ)_2_-AQN are employed to control the enantio- and regioselectivity of the process. According to this pathway, the synthesis of the azpeine ring of (−)-balanol was accomplished in eight steps and in an overall yield of 16%.^82^ More importantly, the two stereogenic centers present in the target natural product 69 were generated *via* a ASAH methodology using modified olefin substrates. The synthesis of azepine core of (−)-balanol commenced from market purchasable 4-chloro-1-butanol which in two steps afforded olefin 73 in 77% overall yield. The ASAH of 73 accomplished as anticipated giving the α-amino-β-hydroxy ester 74 with satisfactory levels of enantioselectivity (82% ee). By ^1^H-NMR data analysis of the crude product, the ratio of regioisomers was determined as >20 : 1. As a result, the hexahydroazepine core 75 was provided in eight steps (from 4-chloro-1-butanol) with an overall yield of 16% (Scheme 16).^82^

**Scheme 17:**
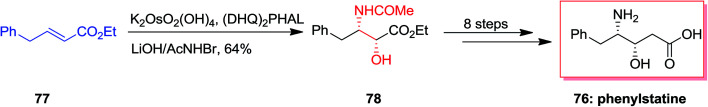


Kumar and co-workers in 2004 developed a stereoselective construction of phenylstatine 76 by using an ASAH reaction as the main step. Therefore, ASAH reaction of α,β-unsaturated ethyl ester 77 using K_2_Os_2_(OH)_2_ as the oxidant reagent, (DHQ)_2_PHAL as a chiral ligand and *N*-bromoacetamide (AcNHBR) as the nitrogen source gave the desired *N*-acetyl derivative 78 in a 10 : 1 regioisomeric ratio and 64% yields with 89% ee, that after some steps afforded phenylstatine 76 (Scheme 17).^83^

**Scheme 18:**
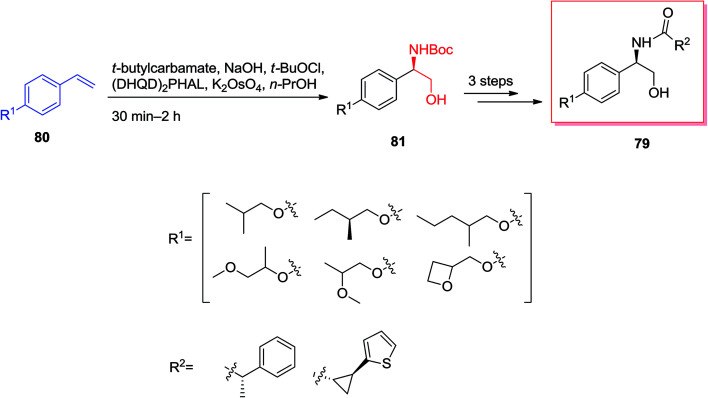


57GPR88 was a member of the G protein coupled receptor (GPCR) showing expressive effect central nervous system and significant influence in peripheral tissues involving liver.^84^ Dzierba and co-workers explored the structure–activity relationships for this superfamilies' by examining the compounds in GPR88 functional analyses.^85^ A series of phenylglycinols and phenyl amines relied on an HTS hit were prepared and examined for potency as agonists of GPR88 by Dzierba and co-workers in 2015. An initial set of biaryl analogs demonstrated moderate agonist property. The common synthesis of the phenylglycinol analogs was shown in Scheme 18. For the synthesis of phenylglycinol 79, ASAH reaction of the vinyl group of 80 gave optically pure Boc-masked amino alcohol 81 that after three steps afforded the phenylglycinol 79. Replacement of the terminal ring of the biaryl group with an aliphatic ether was anticipated to decrease the lipophilicity of the analogs. Therefore, a number of ether analogs has been synthesized by changing the length and branching of the alkoxy group.^85^

**Scheme 19:**
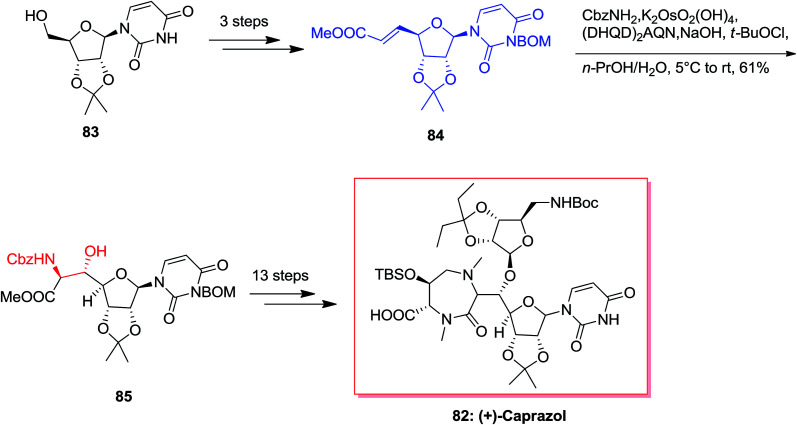


Caprazamycins, lipo-nucleoside antibiotics, are a mixture containing 7 aliphatic side chains that are different in lengths and branched patterns. They were screened showing remarkable anti-tuberculosis (TB) activities.^86^ Among these aliphatic acids, the extracted and isolated prazamycin B showed being the most powerful anti-TB compound, because it can inhibit the action of MraY, an enzyme, responsible for the peptidoglycan biosynthesis.^87^ Total synthesis of caprazol 82 has been initiated from isopropylideneuridine 83 that upon over three steps afforded 84 (*trans*/*cis* = 37 : 1). Next, ASAH reaction of 84 using (DHQD)_2_AQN as a chiral ligand gave 85 with a 5′*S*,6′*S*/5′*R*,6′*R* ratio of 86 : 14. Without the chiral ligand, the reversion of the diastereoselectivity occurred and provided 85 in a ratio of 40 : 60 with a decline in yield. Upon several steps, total synthesis of (+)-82 was accomplished in an extremely short method over 18 steps (Scheme 19).^88^

**Scheme 20:**
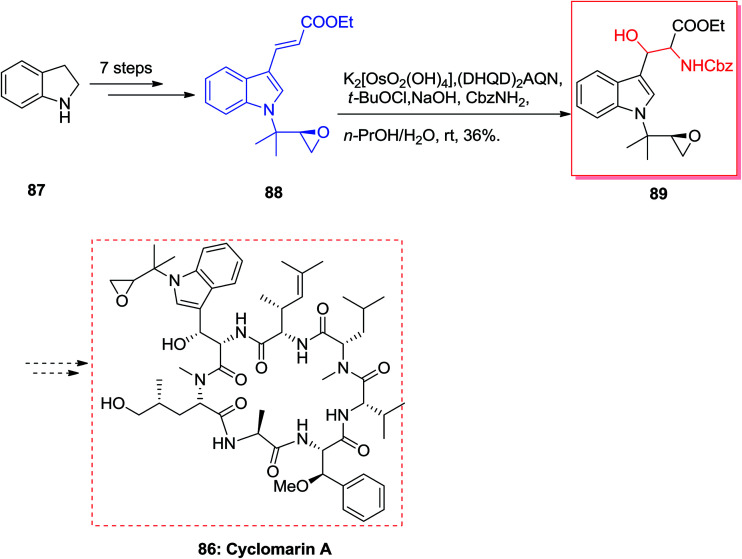


Cyclomarin A 86 that is a cyclic peptide was extracted by Fenical and co-workers from the marine bacterium *Streptomyces* sp.^89^ Cyclomarin A demonstrated significant anti-inflammatory activities in both *in vivo* and *in vitro* assays.^90,91^ Yokokawa and co-workers in 2002 demonstrated an effective approach for the synthesis of uncommon amino acid component 89, which is the key intermediate for the formation of the cyclomarin A 86. Total synthesis of cyclomarin A 86 was started from indole 87 that after seven steps *E*-olefin 88.^92^ Then, the key ASAH reaction of 88 gave the corresponding β-hydroxytryptophan fragment 89 in 36% yields (Scheme 20).^93^

**Scheme 21:**
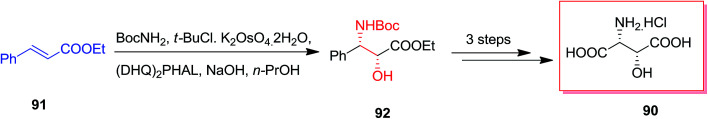


Various stereoisomers of β-hydroxyaspartic acid and their derivatives are realized as the free amino acid and also as peptide components in several fungi and microorganisms.^94,95^ A range of macrocyclic peptide antibiotics, for instance plusbacins,^96^ katanosins,^97^ cepacidine A1,^98^ involve the 3-hydroxyaspartic acid (or, 3-hydroxyasparagine) structural motif in their peptidic scaffold. Because of their potent antibacterial property of this class,^99^ synthesis of them has obtained the attention of chemists.^100,101^ Khalaf and co-workers in 2008, reported a short synthetic method for the synthesis of (2*R*,3*R*)-3-hydroxyaspartic acid 90 in moderate yields (45%). At the first step, ASAH reaction of *trans*-ethyl cinnamate 91 led to the desired *syn*-1,2-amino alcohol 92 in an extremely and stereo- and regio-selective approach. Consequently, acetonide protection, oxidative degradation of the phenyl and removal of the protecting groups provided enantiopure hydrochloride salt of (2*R*,3*R*)-3-hydroxyaspartic acid 90 (Scheme 21).^102^

**Scheme 22:**
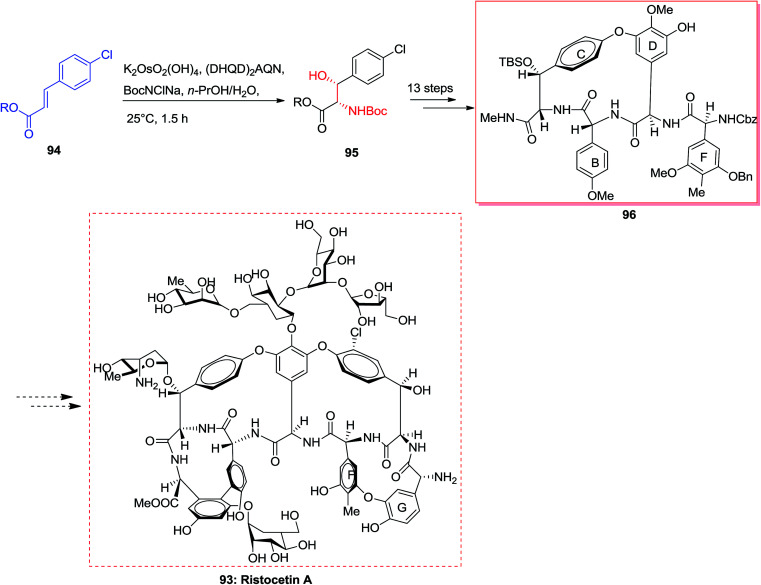


The vancomycin group of antibiotics^103^ are of attention to synthetic chemists because of the structural diversity and the clinical significance of these compounds.^104^ Ristocetin A 93 has structural aspects that are analogous to vancomycin, however includes an additional 14-membered biaryl ether linking between amino acid residues F and G.^105^ In 2001, Pearson and co-workers synthesized the BCD ring system of ristocetin A 96, which was started from the chlorocinnamic esters 94. Significantly, ASAH reaction of chlorocinnamic esters 94 gave directly the *N*-Boc-masked arylserines 95. Next, upon 13 steps, the silyl ether/*N*-methylamide 95 converted to the 16-membered BCD model of ristocetin A 96. Finally, upon several steps, the complicated target compound ristocetin A 93 more reactions was synthesized (Scheme 22).^105^

**Scheme 23:**
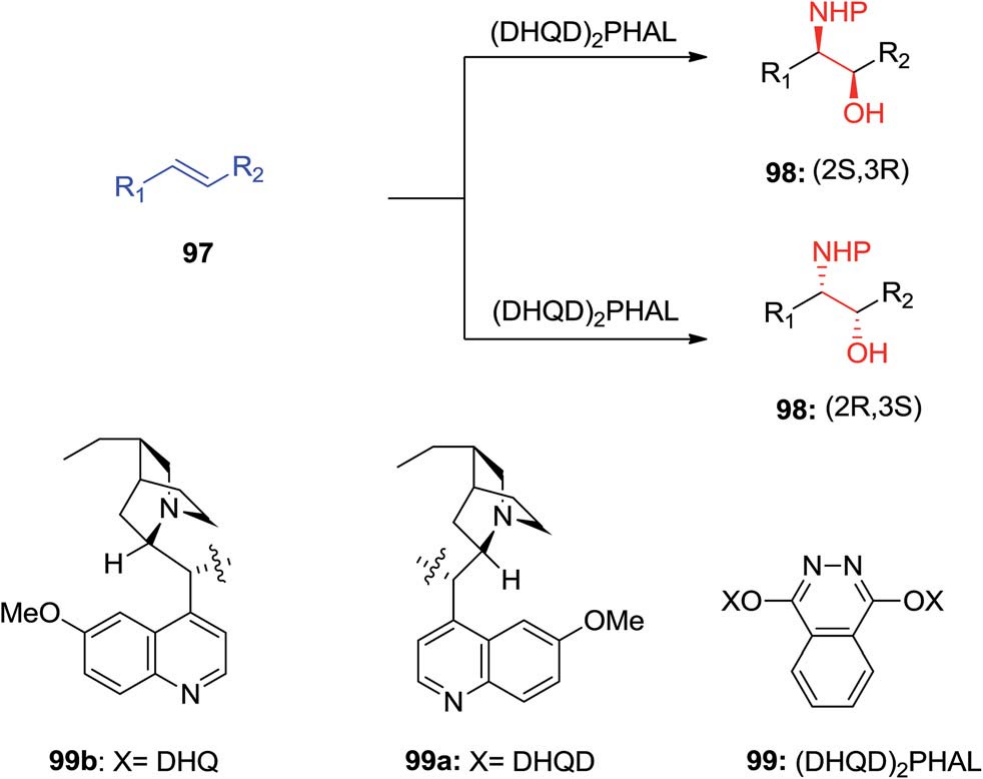


**Scheme 24:**
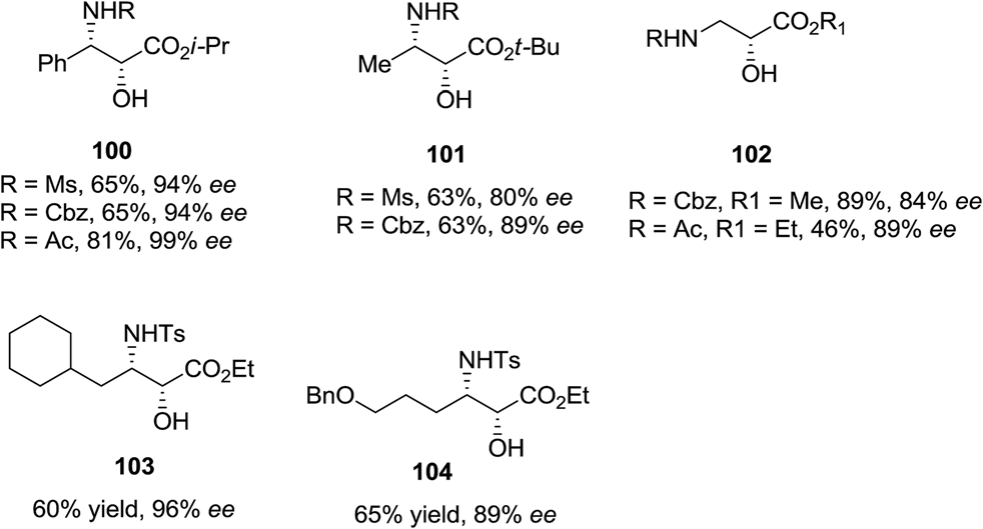


**Scheme 25:**
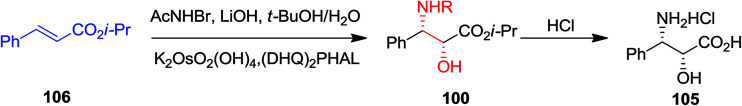


ASAH reaction of olefins is a useful approach for asymmetric synthesis of *N*-masked amino alcohol derivatives. If the substrate was an α,β-unsaturated ester (R^2^ = ester) 97, *syn*-α-hydroxy-β-amino acid 98, a significant pharmacophore realized in various biologically potent products, were provided in enantiopure form (Scheme 23). Commonly, the reaction was occurred in an alcohol/H_2_O mix-solvent by using an alkaloid ligand and a catalytic quantity of K_2_OsO_2_(OH)_4_. Particular results of ASAH reaction of α,β-unsaturated esters to synthesize α-hydroxy-β-amino acid derivatives 100–104 are shown in Scheme 24. Among them, α-hydroxy-β-amino acid 103 was the key constituent of renin inhibitor cyclohexylnorstatine,^106^ and amino acid 104 was the main scaffold of antibiotic Loracarbef.^107^ A significant instance of using ASAH for the formation of natural occurring compounds, was the Sharpless' elegant large-scale construction of the taxol side chain 105.^108^ Upon, two steps, the desired product has been provided in 68% yields and 99% enantioselectivity (Scheme 25).^109^

**Scheme 26:**
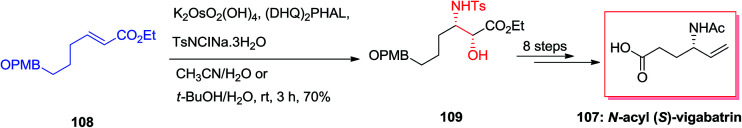


Total synthesis of *N*-acyl (*S*)-vigabatrin 107 as a γ-substituted γ-amino acid was initiated from (*E*)-α,β-unsaturated ethyl ester 108 by Chandrasekhar and co-workers. In this strategy, ASAH reaction of (*E*)-α,β-unsaturated ethyl ester 108 gave the optically enriched amino alcohol 109 with 85% enantioselectivity that upon eight steps yielded the *N*-acyl (*S*)-vigabatrin 107 (Scheme 26).^110,111^

**Scheme 27:**
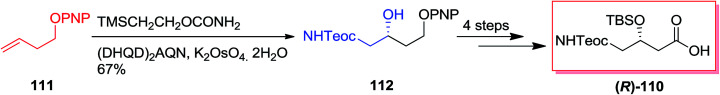


In 2005, Harding and co-workers demonstrated total synthesis of (*R*)-110 as a diprotected (*R*)-γ-aminobutyric acid derivative starting from ether 111. In this strategy, ASAH reaction of ether 111 by using dihydroquinidine ligand (DHQD)_2_AQN gave γ-amino alcohol (*R*)-112 in 81% ee and 67% yields that after four steps produced the diprotected (*R*)-γ-amino-β-hydroxybutyric acid derivative (*R*)-110 in 85% yields (Scheme 27).^110,112^

**Scheme 28:**
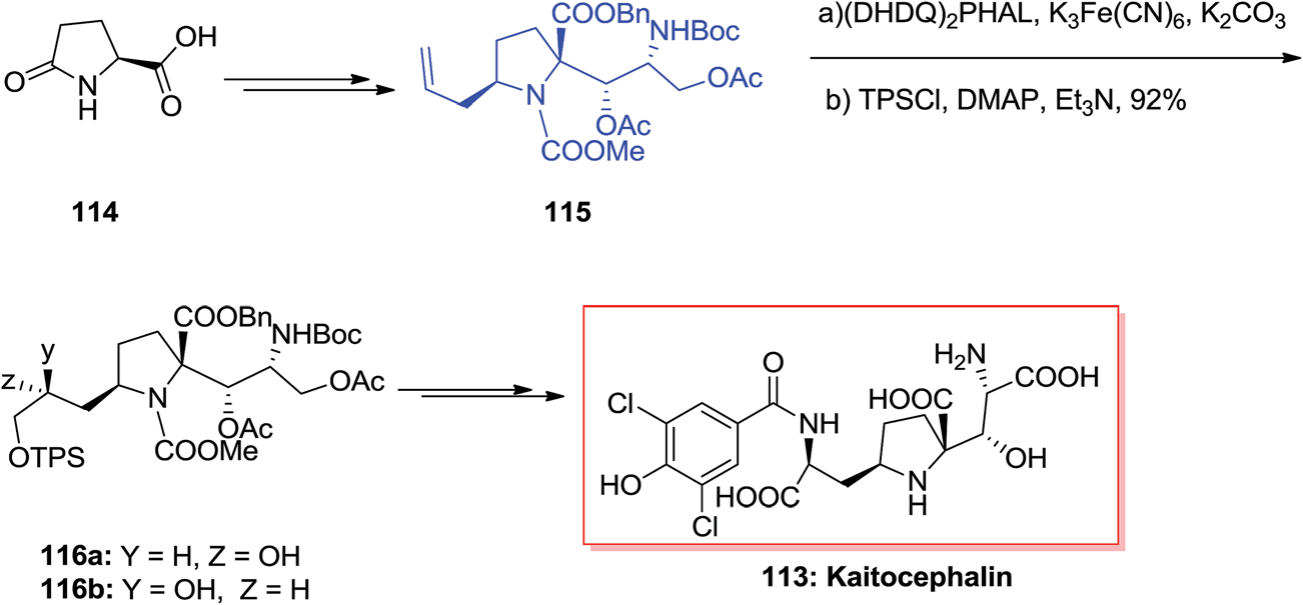


The natural AMPA/KA antagonist, kaitocephalin 113, initially was isolated from the extract of *Eupenicillium shearii*. Using the models of chick primary telencephalic and rat hippocampal neurons, this compound demonstrated protection from kainate toxicity and from AMPA/cyclothiazide. Dissimilar to the previously known AMPA/KA antagonists having a quinoxalinedione moiety, kaitocephalin 113 did not show any cytotoxicity.^113^ Ma and co-workers in 2017 accomplished and reported an efficient total synthesis of kaitocephalin in 25 linear steps, in 8% overall yields.^114^ In this strategy the extremely diastereoselective aldol condensation reaction, ASAH, reduction and Jone's oxidation have played vital roles. Accordingly, the total synthesis of kaitocephalin 113 was initiated from (*R*)-Garner aldehyde 114, which in several steps provided compound 115.^115^ The latter was initially subjected to ASAH, followed by protection of primary hydroxy group with TPSCl to give 116a and 116b in the ratio of 116a/116b 2.2/1. This ratio showed that when commercial AD-mix-β was applied the chiral centers in 115 had some mismatched influence on the diastereoselectivity in the ASAH step. Thus, enriched AD-mix-β, was used to increase the ratio of 116a to 116b to 6.8/1. Finally, compound 116a after several steps was transformed into kaitocephalin 113.^114^ As a matter of fact, the final product 113 was identified to be a mixture of 2-*epi*- and 9-*epi*-2-*epi*-kaitocephalins and other minor isomers and not the pure desired target natural product. Interestingly, natural kaitocephalin 113 was synthesized following the same synthetic method but employing (*S*)-Garner aldehyde instead of its R enantiomer as the substrate in aldol reaction step (Scheme 28).^115,116^

### 3.4. Sugars

**Scheme 29:**
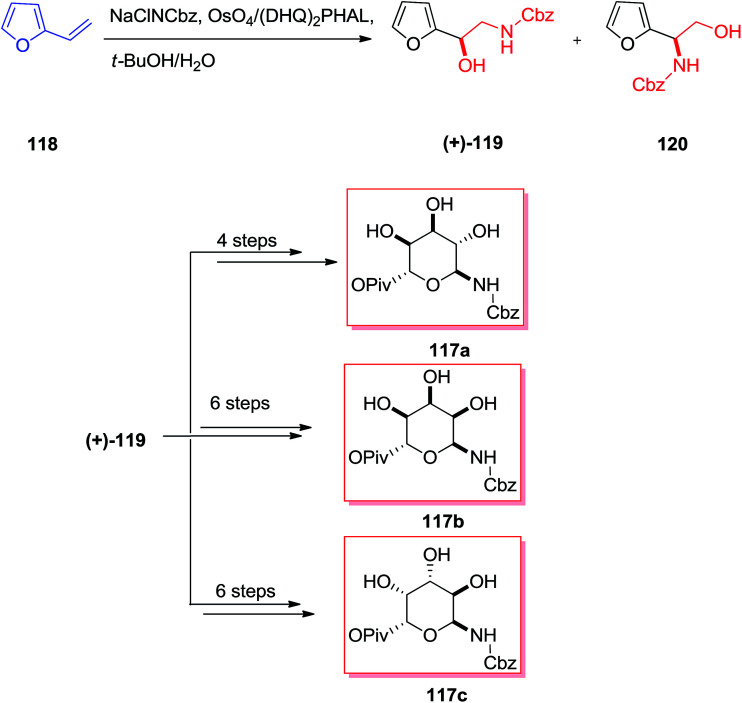


From the identification in the 1950s, the aminoglycoside derivatives have been a significant group of antibiotics in the fight against infections.^117^ Remarkably, the aminoglycoside derivatives involve an excessive group of mono- and bis-glycosidated diaminocyclitol derivatives for example kana-mycins A–C^118^ and so on.^119^ The asymmetric synthesis of three 6-amino-6-deoxy sugar derivatives 117a–c were accomplished in six to eight steps initiating from furfural 118. According to this strategy, a sequence of diastereoselective oxidation reaction and reduction provided Cbz-masked 6-aminomannose from furfuryl alcohol 119. This group demonstrated that *N*-Cbz-masked amino alcohol 119 has been provided in 42% yield as the main regioisomer (2 : 1 ratio) from the ASAH of vinylfuran, although in poor ee. Significantly, by applying the (DHQ)_2_PHAL ligand, the minor isomer (+)-120 has been produced in more than 87% ee, whereas the major isomer (+)-119 was generated with 14% ee. Therefore, the pseudoenantiomeric ligand (DHQD)_2_PHAL afforded the enantiomer (+)-119 in a somewhat increased ee (20%) and (+)-120 in an analogous ee (87%). Although, the application of (DHQ)_2_AQN as a ligand in the ASAH was demonstrated to accomplish a reversal of regioselectivity, its application in the ASAH of vinylfuran provided results analogous to those of (DHQ)_2_PHAL. Finally, the corresponding 6-amino-6-deoxy sugars 117a–c were synthesized upon several steps (Scheme 29).^120^

**Scheme 30:**
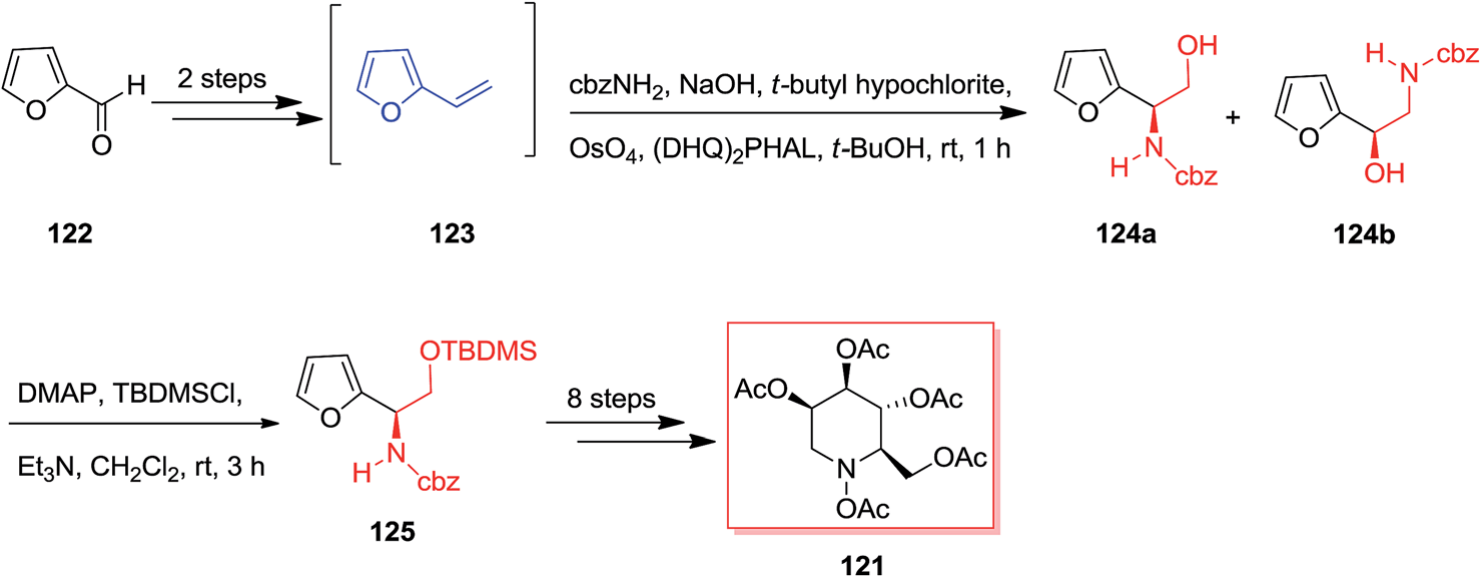


Both Ciufolini group in 1998^121^ and O'Doherty group in 2001^122^ have reported an azasugar synthesis through an ASAH reaction/aza-Achmatowicz method. According to O'Doherty's strategy, furfural 122 using a Grignard reaction and then by the addition of 1 M hydrochloric acid provided 2-vinyl-furan 123. Enantiomerically improved *N*-Cbz-masked amino alcohol derivatives 124a and 124b were provided through the ASAH reaction of 123. This was accomplished by reacting furan 122 with the sodium salt of *N*-chlorobenzylcarbamate and a osmium tetroxide/(DHQ)_2_PHAL mixture (AD-mix-α). The highest ee was provided with the (DHQ)_2_PHAL ligand system, that afforded 125 in a 21% yield from furfural 122 (>86% ee). In the following, regioisomers 124a and 124b have been generated in a 1 : 2 ratio and also were inseparable at this step. Finally, purification of 124a and additional change in a 10-step route resulted in the penta-acetate 121 (Scheme 30).^123^

**Scheme 31:**
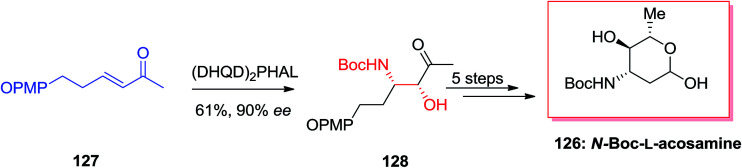


Amino sugars play a major role in the overall carbohydrate field, and they show particular challenges for the synthetic chemist pursuing to effect valuable and controllable conversions in the direction of targets of biological significance.^124^ McLeod and co-workers optimized ligand/substrate control of regioselectivity for the synthesis of natural products 3- and 4-aminosugars.^125^ For this purpose, initially, β-aminoketone precursor 128 obtained from the α,β-unsaturated methyl ketone 127*via* the agency of (DHQ)_2_PHAL catalyzed ASAH reaction in 90% ee and 61% yield. Finally, after several steps, *N*-Boc-l-acosamine 126 was synthesized in 18% overall yields (Scheme 31).^125^

**Scheme 32:**
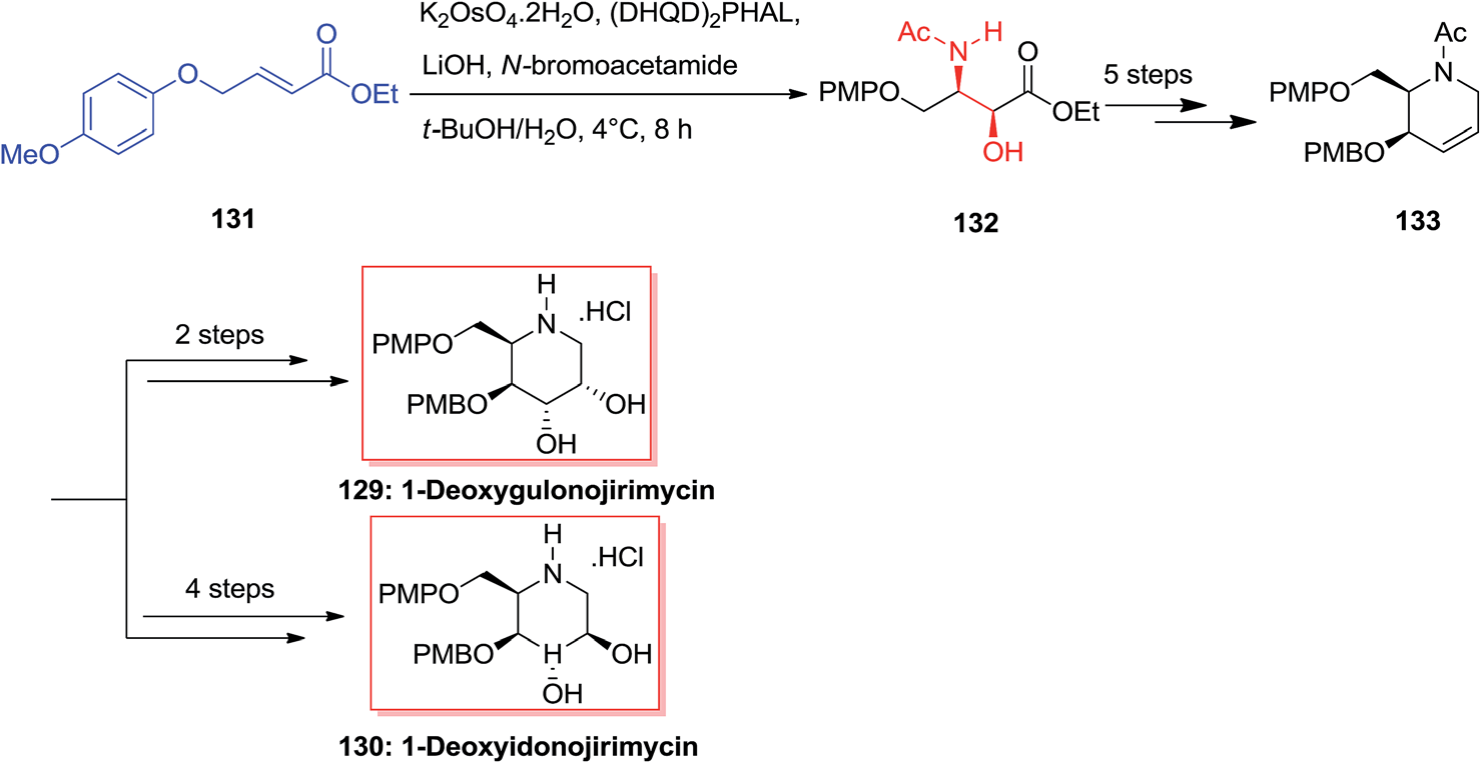


A common synthesis of 1-deoxyazasugar derivatives has been achieved by Singh and Han in 2003,^126^ through the general olefin intermediate 133. The formation of the key compound was started from olefin 131 (easily synthesized from *p*-methoxyphenol and 4-bromocrotonate). The aryl groups have been selected, as it was assumed that aryl–aryl stacking interactions between 131 and the ASAH reaction would advance with increased selectivity. Actually, the reaction was accomplished with high regioselectivity (>20 : 1) to provide the amino alcohol 132. After several steps olefin 133 was provided as a key intermediate that has been applied to synthesize 1-deoxymannonojirimycin 129 and 1-deoxyidonojirimycin 130 (Scheme 32).^126^

### 3.5. Lactone and lactams

**Scheme 33:**
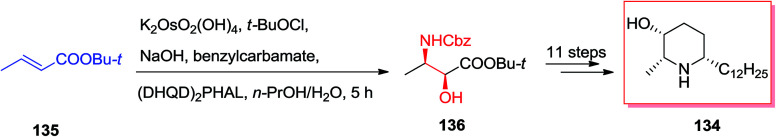


In year 2006, Kumar and co-workers demonstrated an extremely effective pathway for the production of substituted piperidine derivatives that are among the most abundant heterocyclic frameworks in naturally occurring compounds and synthetic products with significant properties. Total synthesis of (−)-deoxocassine 134, a *cis*-2,6-disubstituted 3-piperidinol, was started from market purchasable *t*-butyl crotonoate 135. Initially, compound 135 has been exposed to ASAH reaction by utilizing benzyl carbamate as a nitrogen source, K_2_[OsO_2_(OH)_4_] as an oxidant, and (DHQD)_2_PHAL as a chiral ligand to form the amino alcohol 136 in excellent regio- and enantio-selectivity. Several more steps required to accomplish the desired natural product (−)-deoxocassine 134 (Scheme 33).^127^

**Scheme 34:**
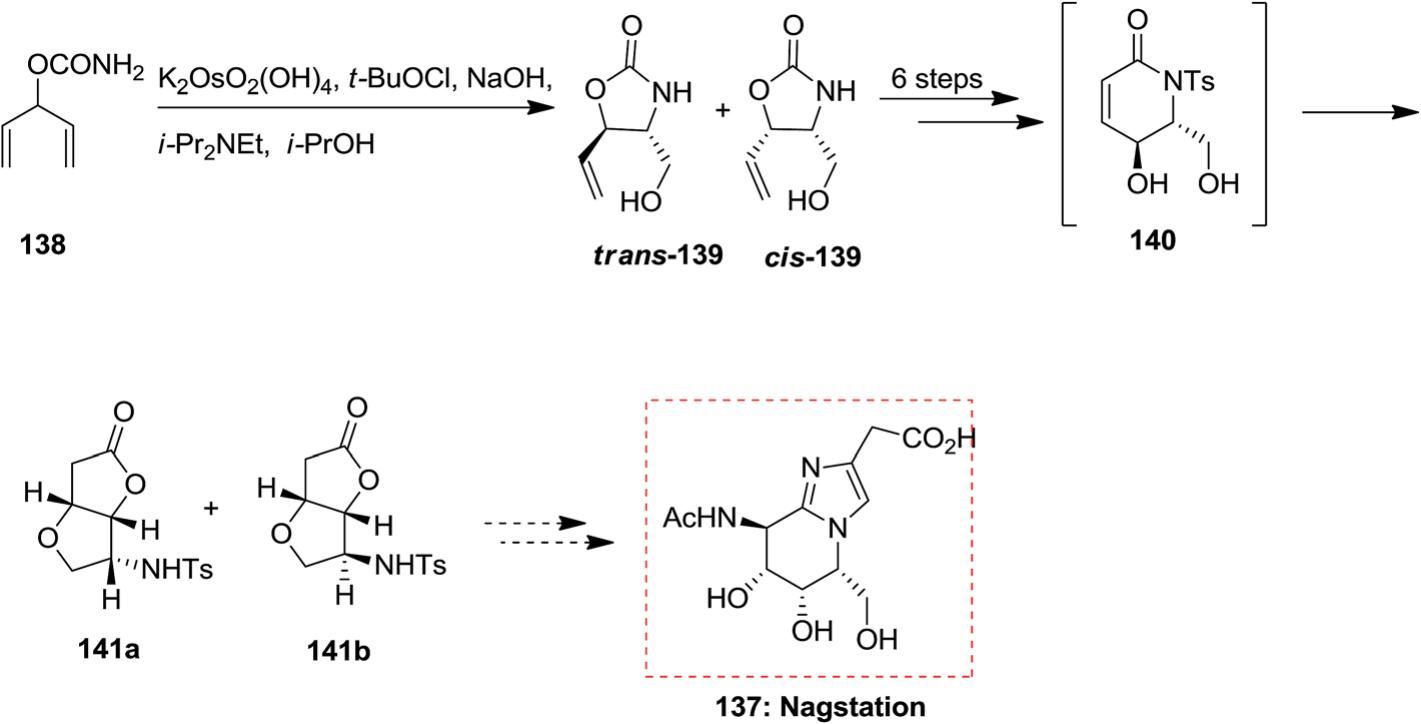


A variant of Knight's method to d-mannolactam, exploring the stereoselectivity of directed oxidation condition reactions, demonstrates a tendency for hydroxylated *N*-tosyl lactam derivatives to rearrange to γ-lactone derivatives. Studies toward the total synthesis of nagstation 137 was started from carbamate 138 that through ASAH strategy produced oxazolidinone 139 in 65% unoptimized yields. Next, after several steps, including an intramolecular transacylation and 1,4-*O*-addition, the bicyclic lactone 140 was provided along with a small quantity of diastereomer 141b. Although, the timing of procedures to form 141a is open to question (Scheme 34).^128^

**Scheme 35:**
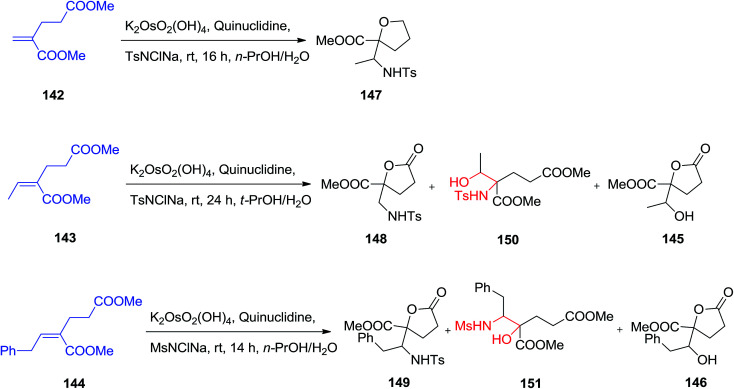


Substituted γ-lactone derivatives have appealed significant interest in recent years because of their significance as building blocks in the formation of a range of naturally occurring products and biologically significant compounds,^129^ for instance, precursors of inhibitors of HIV-1 protease.^130^ SAD and ASAH reaction of (*E*)-dimethyl-2-alkylidene glutarates 142–144 were displayed to afford enantio-enriched or enantiopure highly functionalized γ-butyrolactone derivatives 145, 146 and 147–149. The regioselectivity of the ASAH reaction has been controlled by different parameter, such as alkene polarization, alkene substitution, and ligand–substrate interactions.^131^ It was well developed that the nitrogen group was usually introduced at the β-position in α,β-unsaturated esters.^132^ Dimethyl-2-methylene glutarate 142 was exposed to the ASAH manner, by using marketably accessible chloramine-T as a nitrogen source, the resulting α-hydroxy isomer was provided as the major compound and lactonized unexpectedly to yield the desired substituted γ-butyrolactone 147 in satisfactory yield, but with low to satisfactory ee (19–63% ee). The ASAH of 143 using quinuclidine as ligand afforded the γ-butyrolactone 148 in 30% yield accompanied by the β-hydroxy regioisomer 150 in 6%, the dihydroxylation product 145 (26%) and recovered initiating compounds (25%).^133^ To improve the selectivity, it was tried as a nitrogen source, chloramine-M, which is less sterically hindered than chloramine-T. Fascinatingly, it was known that catalytic amino-hydroxylation reaction of 144 resulted mostly in the α-hydroxy regioisomer 151 (54% yields) accompanied by the lactonized product 149 (22%), the lactone 146 (18%) generated using competitive dihydroxylation as well as some recovered starting compound (5%) (Scheme 35).^133^

### 3.6. Miscellaneous

**Scheme 36:**
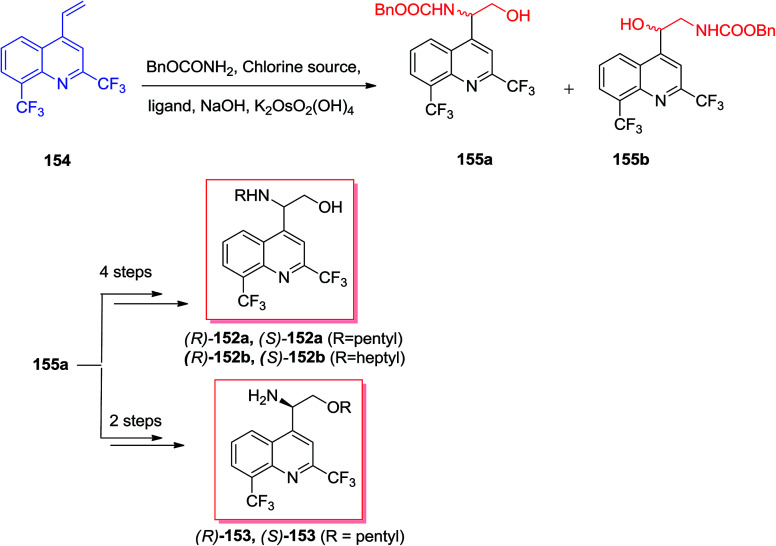


Sonnet and co-workers exhibited enantioselective synthesis of antimalarial aminoquinolines *via* ASAH reaction in 2016.^134^ Aminoquinolinethanols (*R*)-/(*S*)-152 and quinolinethanamines (*R*)-/(*S*)-153 was prepared and antimalarial property of them was explored. In this strategy, the ASAH reaction of 4-vinylquinoline 154 have been accomplished by using osmium(vi) pre-catalyst, potassium osmate(vi) dehydrate and ligand (DHQ)_2_PHAL or (DHQD)_2_PHAL. Therefore, conditionally, the majority of compound provided was allocated as the (−)-(*S*)-155 once (DHQ)_2_PHAL has been used and (+)-(*R*)-155 once (DHQD)PHAL was applied. Next, after several steps, the enantiomers 155 gave three series of enantiopure aminoquinolines 152a, 152b, and 153 (Scheme 36).^134^

**Scheme 37:**
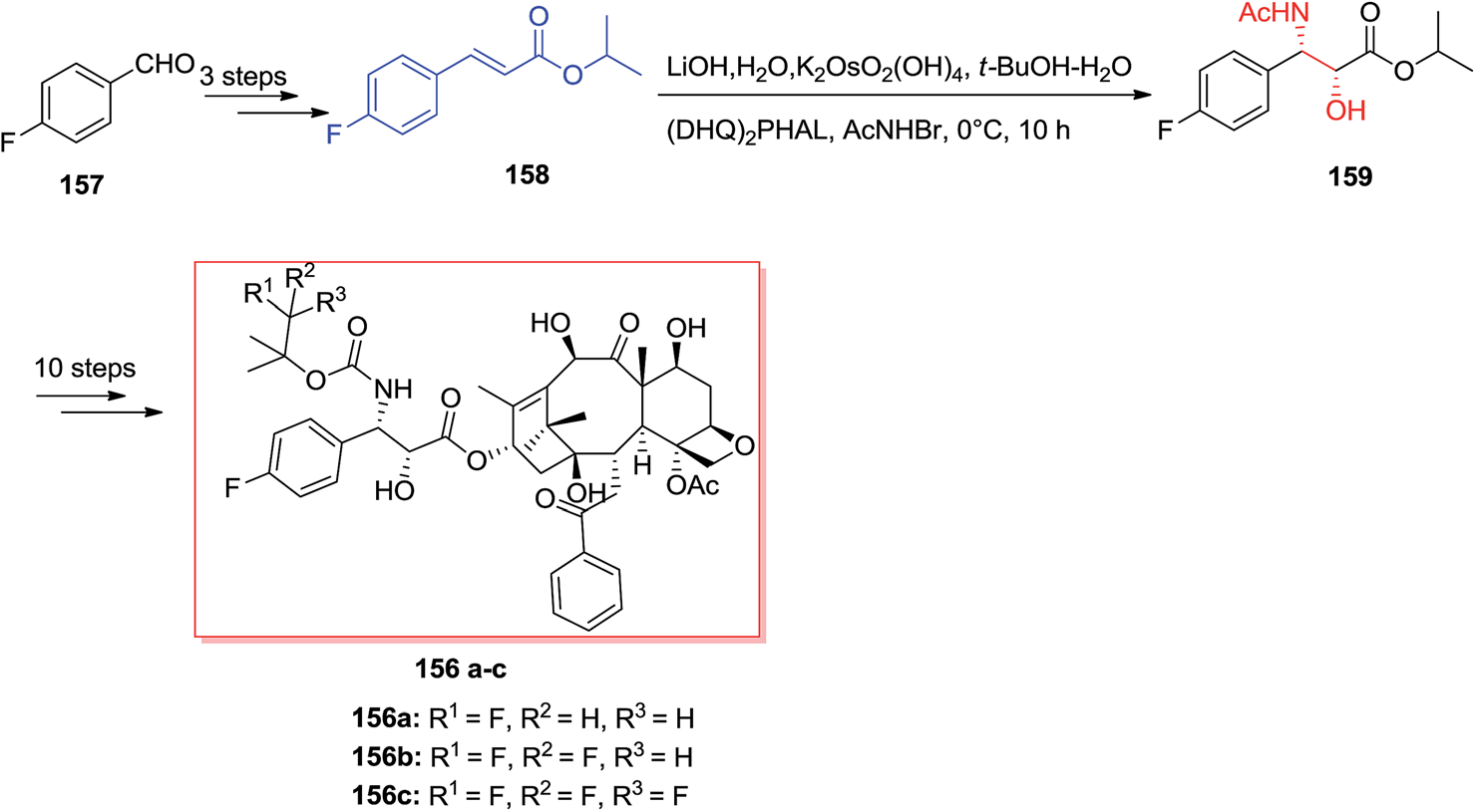


The famous natural product taxol a diterpenoid extracted from the bark of *Taxus brevifolia*.^135^ Nowadays, it can be bio-synthesized by microorganisms and semi-synthesis. Paclitaxel and its semi-synthetic derivative docetaxel are currently known as the most significant and auspicious anticancer especially for breast and ovarian cancers because of their distinctive mechanism of action by binding tubulin and stabilizing microtubule construction, that finally interrupts mitosis resulting in cell destruction.^136^ Three significant fluorine-comprising docetaxel analogs 156a–c have been prepared by Sun and co-workers in 2011.^137^ Total synthesis of products 156a–c was started from 4-fluorobenzaldehyde 157, that after several steps, afforded isopropyl cinnamate 158. Significantly, isopropyl cinnamate 158 was exposed to a ASAH reaction by applying (DHQ)_2_PHAL as the ligand to provide the corresponding amino alcohol 159 as a single isomer with >99% ee and in 83% yields. Lastly, compound 159, after several steps, yielded the desired products 156a–c (Scheme 37).^137^

**Scheme 38:**
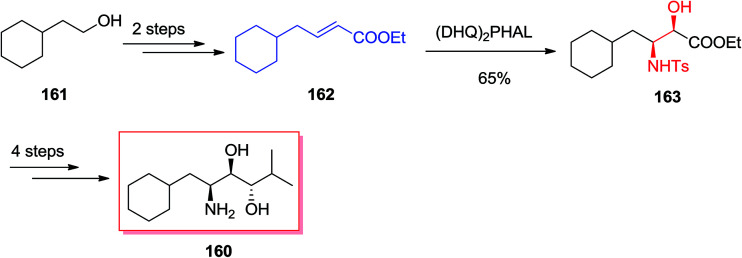


Renin, as an aspartic protease, was found to be the rate-determining enzyme in the cascade resulted in the vasopressor substance angiotensin-II, which shows a vital activity for the regulation of blood pressure. It was demonstrated that inhibitors such as Zankiren and Enalkiren include a core unit so-called the Abbott amino-diol (2*S*,3*R*,4*S*)-2-amino-*l*-cyclohexyl-6-methyl heptane-3,4-diol 160. Chandrasekhar A and co-workers reported a concise and useful enantioselective approach for the synthesis of Abbott amino-diol 160. In five steps by using ASAH reaction as the key step starting from market purchasable cyclohexyl ethanol 161.^138^ In this route, total synthesis of Abbott amino-diol 160 was started from cyclohexyl ethanol 161, that after two steps provided α,β-unsaturated ester 162. Next, ester 162 was exposed to SAH by using catalytic quinine obtained ligand (DHQ)_2_PHAL and K_2_OsO_2_(OH)_4_ in *t*-BuOH/H_2_O (1 : 1) to make (2*R*,3*S*)-*N*-(*p*-toluenesulphonyl)-3-amino-4-cyclohexyl-2-hydroxy butyrate 163 in 65% yield and 89% ee. Several more steps required to be done in order to accomplish the desired product 160 (Scheme 38).^138^

**Scheme 39:**
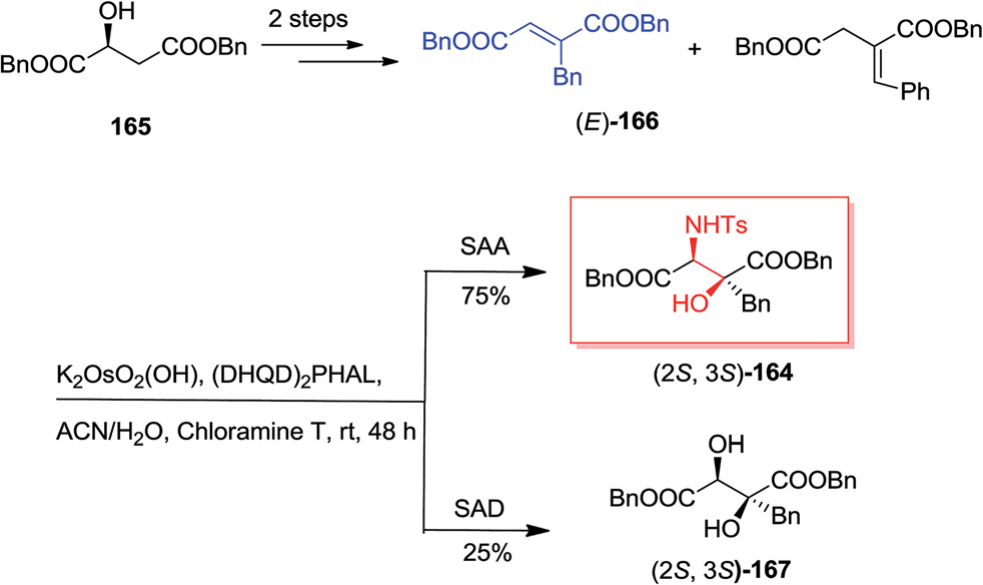


Because of the biological property of β-alkyl-β-hydroxyaspartates as significant blockers of glutamate transporters (EAATs) impacting on glutamatergic synapses activity, Rolland and co-workers demonstrated a short, enantioselective synthesis of enantiomerically pure *threo*-β-benzyl-β-hydroxyaspartates.^139^ Remarkably, the key stage was a stereoselective and regiospecific ASAH reaction on benzyl fumarate. In this manner, synthesis of amino alcohol 164 was initiated from market purchasable (*S*)-malic acid 165 that after some steps gave (*E*)-dibenzyl 2-benzylfumarate 166 in satisfactory yields.^140^ Next, the dehydration can be occurred on racemic dibenzyl 2-benzyl-3-hydroxysuccinate. Also, the construction of dibenzyl 2-benzylidene succinate regioisomer in 20% yields was because of uncontrolled double bond isomerization. In the following, 10 mol% of (*E*)-benzylfumarate 166 has been used in the first catalytic cycle of the reaction and upon 30 min of stirring, the residual 90 mol% of (*E*)-benzylfumarate 166 has been added and provided the second catalytic cycle. This efficient method gave a 25 : 75 ratio of diol 167 to amino alcohol 164 (Scheme 39).^139^

**Scheme 40:**
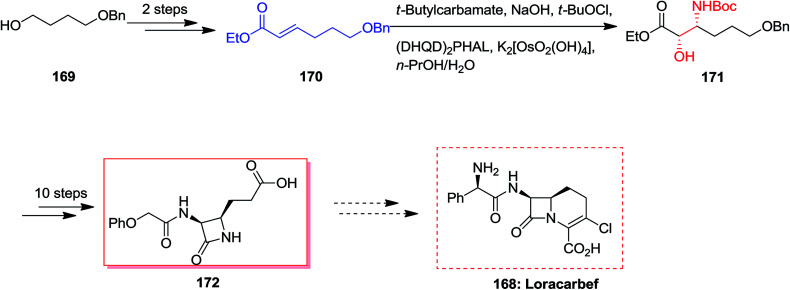


Loracarbef 168 was a carbacephalosporin antibiotic having the extended chemical and serum stability.^141^ A formal synthesis of loracarbef 168 was demonstrated by Kang and co-workers in 2001.^107^ In this strategy, formal synthesis of *cis*-3,4-disubstituted azetidinone 172 was initiated from monoprotected 1,4-butanediol 169 that after two steps resulted in the synthesis of α,β-unsaturated ester 170.^107^ In the succeeding, the ASAH reaction of 170 with the sodium salt of *t*-butylcarbamate and a mixture of K_2_[OsO_2_(OH)_2_]/(DHQD)_2_PHAL afforded the corresponding regioisomer [2*S*,3*R*]-171 in satisfactory yields with excellent regioselectivity (>13 : 1) and 89% ee. Lastly, several more steps was used to generate the corresponding *cis*-3,4-disubstituted azetidinone 172 (Scheme 40).^107^

**Scheme 41:**
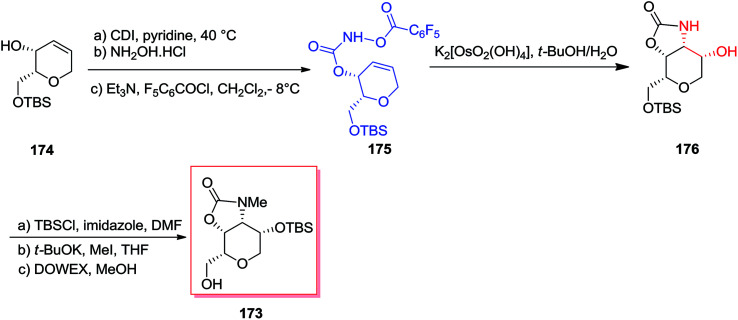


A short stereoselective method for the formation of dysiherbaine tetrahydropyran unit has been developed in nine steps and 39% overall yield by Carroll and co-workers in 2011.^142^ Donohoe's advanced tethered aminohydroxylation conditions were used to simultaneously establish the amino and alcohol groups and made the tetrahydropyran ring that shows four contiguous *cis*-stereocenters. Total synthesis of the target product 173 was started from the allylic alcohol 174, which after several steps gave the desired *O*-functionalized hydroxy carbamate 175. After mixing carbamate 175 with potassium osmate, clean transformation to the oxazolidinone 176 has been achieved. Satisfactorily, the improved sodium hydroxide free condition reaction afforded the oxazolidinone 176 as a single isomer, and without any detected migration of the cyclic carbamate that demonstrated detrimental once using the standard TAH conditions. Finally, three more steps required to prepare alcohol 173 in 39% overall yield (Scheme 41).^142^

**Scheme 42:**
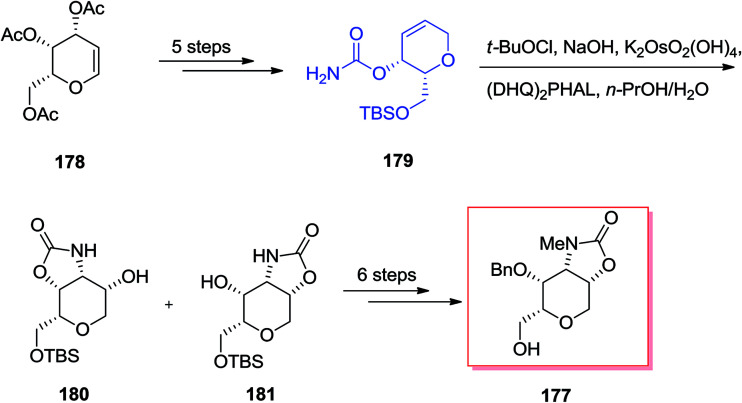


Chamberlin and co-workers demonstrated a stereocontrolled synthesis of an improved intermediate of the dysiherbaine tetrahydropyran unit 177 that was accomplished in 11 steps and 27% overall yield.^143^ Significantly, the main aspect of this synthetic method is the usage of the Donohoe tethered aminohydroxylation reaction to make the amino diol and providing the four contiguous *syn* stereocenters on the tetrahydropyran ring. Total synthesis of the dysiherbaine tetrahydropyran unit 177 has been initiated from tri-*O*-acetyl-galactal 178, which after several steps yielded the carbamate 179. In the following, the significant tethered aminohydroxylation reaction of carbamate 179 led to a 1 : 1 mixture of hydroxy oxazolidinone isomers 180 and 181 (in the crude reaction mixture). Next, several more steps required to give the dysiherbaine tetrahydropyran unit 177 (Scheme 42).^143^

**Scheme 43:**
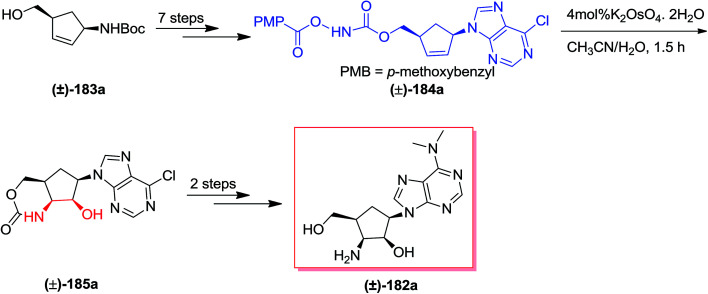


Puromycin, an aminonucleoside naturally occurring compound, shows an extensive range of antitumor and antibiotic properties.^144^ In 2010, Miller and co-workers reported the construction of carbocyclic aminonucleoside derivatives and *epi*-4′-carbocyclic puromycin using an acylnitroso obtained hetero Diels–Alder cycloadduct. Pd(0)/InI-catalyzed allylations of a formyl species were applied to make the 4′-hydroxymethyl group. A tethered aminohydroxylation approach has been used to provide the *cis*-2′,3′-amino alcohol scaffold with full diastereo- and regiocontrol.^145^ The total synthesis of lyxo-carbocyclic aminonucleoside (±)-182 was started from *syn*-1,4 scaffold (±)-183a, which after seven steps afforded the *N*-pentafluorobenzoyloxy carbamate (±)-184. Subsequent, a *t*-BuOH/H_2_O (3 : 1) solution of homoallyl *N*-pentafluorobenzoyloxy carbamate (±)-184 has been reacted with catalytic K_2_OsO_4_, and provided hydroxyamination product (±)-185. Although, two challenging side reactions donated to the low transformation to (±)-185. Finally, the *lyxo*-carbocyclic aminonucleoside (±)-182 were produced upon two steps (Scheme 43).^145^

**Scheme 44:**
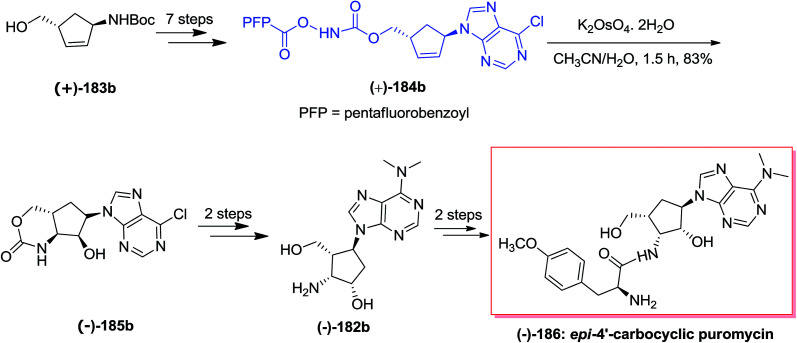


In a similar strategy, *anti*-1,4 diastereomer (+)-183b made the tethered aminohydroxylation precursor (+)-184b. For the aminohydroxylation, homoallyl *N*-pentafluorobenzoyloxy carbamate (+)-184b has been reacted with catalytic K_2_OsO_4_ in acetonitrile/water (1.5 : 1) to yield hydroxyamination product (−)-185b in 83% yield. In this situation, remarkably, the 1′-nucleobase is orientated on the (another) opposite side of the tether and the decreased steric bulk can contribute to the increased yield of (−)-185b in comparison with the reaction of diastereomer (±)-184a. In the following, carbocyclic aminonucleoside (−)-182b has been produced through installation of the dimethylamino scaffold followed by removal of the cyclic carbamate with lithium hydroxide to construct the carbocyclic derivative (−)-182b. Lastly, after two steps, desired carbonucleoside, *epi*-4′-carbocyclic puromycin (−)-186 has been provided in moderate yields (Scheme 44).^145^

**Scheme 45:**
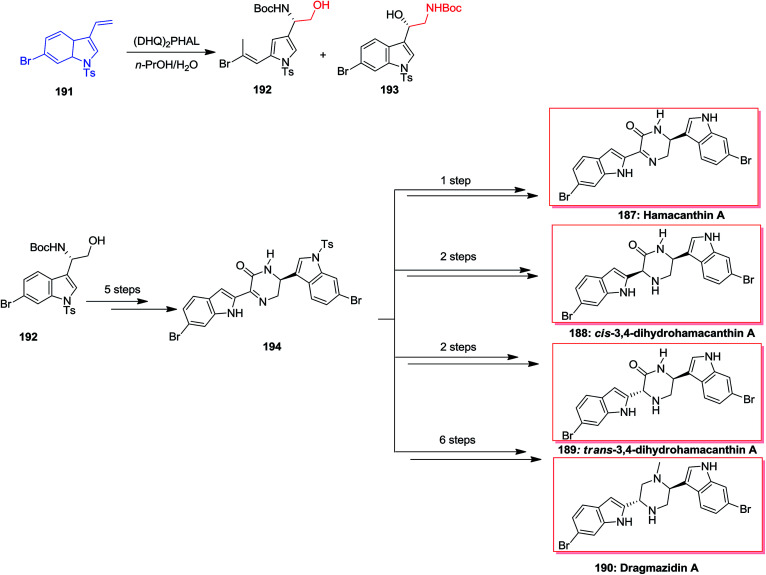


In 2002, Jiang and co-workers developed a convenient method for the direct asymmetric synthesis of (*S*)–*N*-Boc-masked-indol-3-ylglycinols from vinyl indole derivatives by using the ASAH reaction, with 65% of up to yields and ee of up to 94% in 27 steps. Initially, the vinyl 6-bromoindole 191 has been transformed to (*S*)-indolylglycinol 192 and 193 based on the ASAH conditions. In the following, (*S*)-indolylglycinol 192, after several steps the desired intermediate 194, that resulted in the total synthesis of natural products hamacanthin A 187, bisindole alkaloids dihydrohamacanthin *cis*- or *trans*-3,4-dihydrohamacanthin A 188, 189 and dragmacidin A 190 (Scheme 45).^146^

**Scheme 46:**
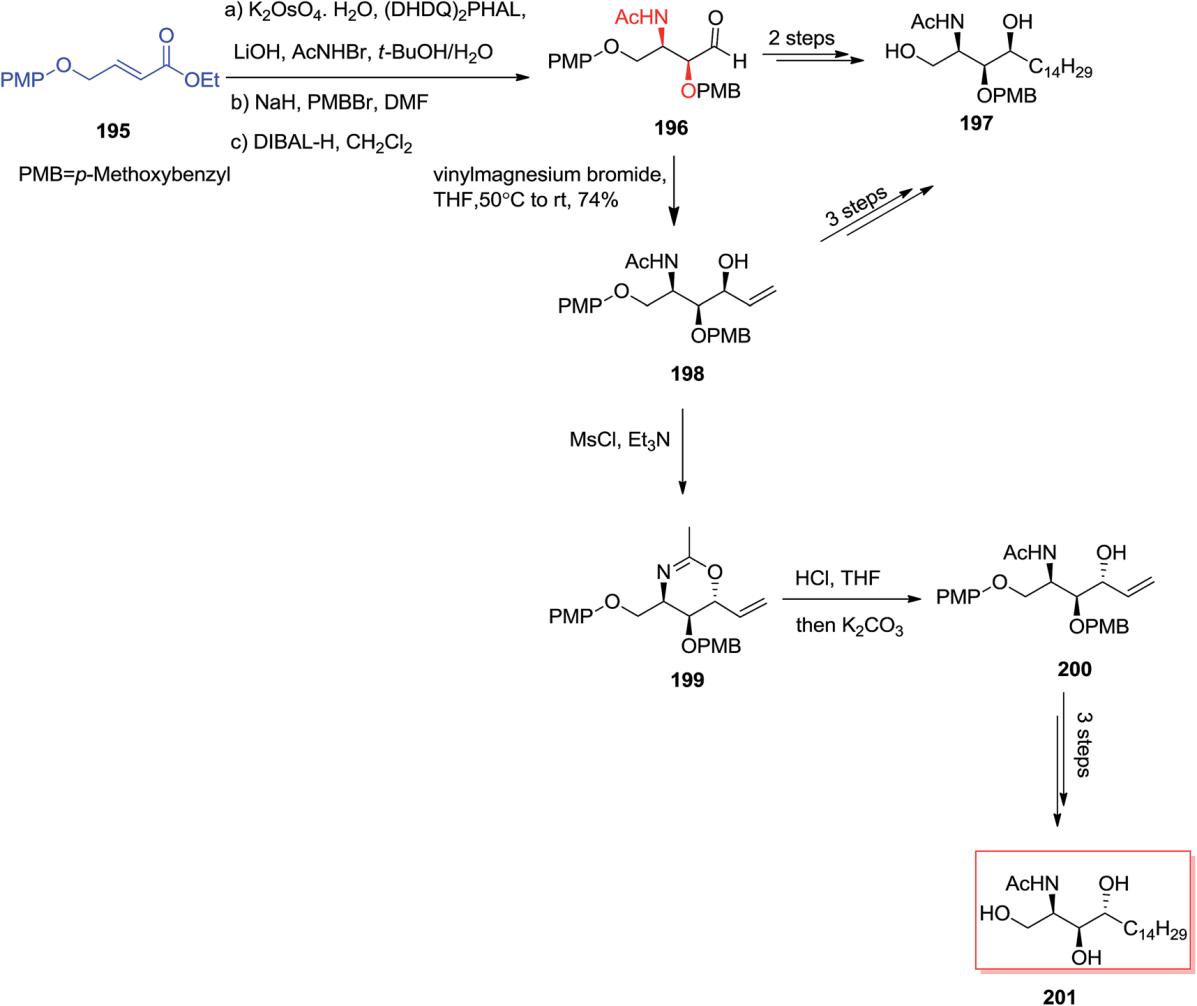


In 2004, Han and co-workers^147^ utilized an ASAH reaction of α,β-unsaturated ester 195 to form the stereocenters at C2 and C3 with high enantio- (>99%) and regioselectivity (>20 : 1). The chirality at C4 (>10 : 1) was provided by an effective diastereoselective addition of aldehyde 196 to an appropriate Grignard reagent. In this line, *N*-acetyl-l-*xylo*-phytosphingosine 197 was produced in five steps in 22% overall yield. Significantly, an alternative synthesis of 197 was also accomplished *via* a two-step manipulation of 198. Accordingly, the stereoselective interconversion of the OH group at C4 has been occurred *via* the reaction of 198 with MsCl/Et_3_N through oxazine intermediate 199 that could further transformed into *N*-acetyl-l-arabino-phytosphingosine 201 (Scheme 46).^147^

**Scheme 47:**
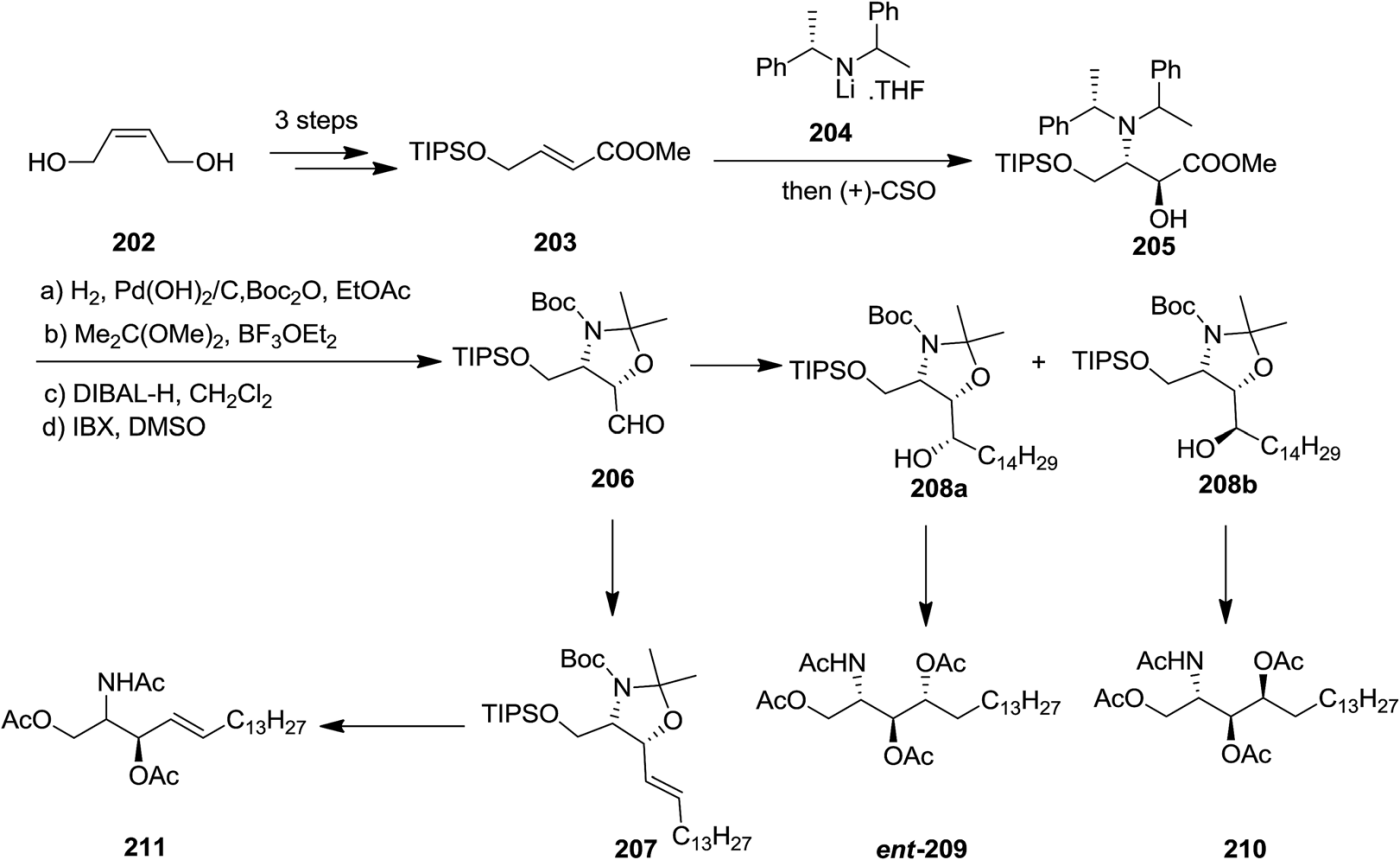


In 2008, the Davies and co-workers reported a divergent and efficient synthesis of *N*,*O*,*O*-triacetyl-d-*erythro*-sphingosine 211, tetraacetyl-d-*lyxo*-phytosphingosine *ent*-209, and tetraacetyl-d-ribo-phytosphingosine 210 from the same intermediate oxazolidine aldehyde 206.^148,149^ Wittig olefination of oxazolidine aldehyde 206 afforded compound 211 with satisfactory *E*-selectivity (*E*/*Z* = 94 : 6) by quenching the reaction with MeOH. Alternatively, addition of 206 to an appropriate Grignard reagent provided a 90 : 10 mixture of alcohols 208a and 208b, that were subsequently transformed into compounds *ent*-209 and 210, respectively. The advantages of this approach were the extremely diastereoselective conjugate addition of unsaturated ester 205 with subsequent *in situ* enolate oxidation with (camphorsulfonyl)oxaziridine (CSO) (Scheme 47).^149^

## 4. Conclusion

In this report, we tried to introduce Asymmetric Sharpless Aminohydroxylation, (ASAH) as one of the most significant, vital and efficient reactions in the total synthesis of naturally occurring compounds, complex molecules with high biological activities as well as applied molecular targets. It should be mentioned that in 2002 ASAH reaction has been reviewed in general term by McLeod *et al.*^15^ Nevertheless, it lacks the applications of this important and key reaction in total synthesis of natural products and complicated compounds. Since then vast developments have been accomplished in ASAH reaction, which was initially disclosed by Sharpless *et al.*, in 1996. The ASAH reaction is very important in total synthesis of natural products with several definite stereogenic centers, which must be induced during their multistep synthesis. ASAH reaction allows the catalytic and enantioselective synthesis of protected vicinal amino alcohols, in a single step, from a broad range of simple alkenes as commercially available or easily accessible starting materials. The importance of this discovery was directly apparent to many organic chemists from synthetics point of view, since the ASAH reaction offers direct access to the array of amino alcohols existed in a wide range of biologically potential agents and natural products. The AH reaction allows the *syn*-selective synthesis of 1,2-amino alcohols *via* reaction of alkenes with salts of *N*-halosulfonamides, -amides and -carbamates using OsO_4_ as a catalyst and oxidizing agent. Enantioselectivity, Sharpless and co-workers achieved AH reaction *via* adding the dihydroquinine- and dihydroquinidine-derived chiral ligands name it as ASAH.

Remarkably, in spite of the enormous potential of ASAH reaction, in the first years of introduction, only relatively some researchers demonstrated interest to develop such a significant synthetic method. In addition, it was mostly overlooked to be applied as a main step in the total synthesis of naturally occurring compounds. Possibly, this lack of interest was because of the challenging the problem of controlling of both the enantio- and regioselectivity of the ASAH reaction. Although, in recent years, different efficient routes for circumventing such problems were examined and opened novel pathway for the effective and simple synthesis of amino alcohols being employed as precursors in a key step of the complex molecules and natural products containing these important scaffolds. In continuation of our previous reports dealing with the two other Sharpless achievements, ASE and ASDH, herein we literally underscored all applications of his another important asymmetric strategy resulting in high stereoselective aminohyroxylation to give optically pure amino alcohols, which are an important and reactive intermediates as one of the key steps in the total synthesis of natural products as well as other important complicated compounds showing biological activities. We hope this report attracts the attention and stir up the interest of synthetic organic chemists to consider this useful strategy as in their future endeavors in designing protocols for total synthesis of natural products.

## Conflicts of interest

There are no conflicts to declare.

## Supplementary Material
